# High-Resolution HLA Typing of HLA-A, -B, -C, -DRB1, and -DQB1 in Kinh Vietnamese by Using Next-Generation Sequencing

**DOI:** 10.3389/fgene.2020.00383

**Published:** 2020-04-30

**Authors:** Minh Duc Do, Linh Gia Hoang Le, Vinh The Nguyen, Tran Ngoc Dang, Nghia Hoai Nguyen, Hoang Anh Vu, Thao Phuong Mai

**Affiliations:** ^1^Center for Molecular Biomedicine, University of Medicine and Pharmacy at Ho Chi Minh City, Ho Chi Minh City, Vietnam; ^2^Faculty of Public Health, University of Medicine and Pharmacy at Ho Chi Minh City, Ho Chi Minh City, Vietnam; ^3^Department of Physiology, Pathophysiology and Immunology, Faculty of Medicine, University of Medicine and Pharmacy at Ho Chi Minh City, Ho Chi Minh City, Vietnam

**Keywords:** high-resolution, HLA typing, allele frequency, haplotype frequency, Kinh Vietnamese, next-generation sequencing

## Abstract

Human leukocyte antigen (HLA) genotyping displays the particular characteristics of HLA alleles and haplotype frequencies in each population. Although it is considered the current gold standard for HLA typing, high-resolution sequence-based HLA typing is currently unavailable in Kinh Vietnamese populations. In this study, high-resolution sequence-based HLA typing (3-field) was performed using an amplicon-based next-generation sequencing platform to identify the HLA-A, -B, -C, -DRB1, and -DQB1 alleles of 101 unrelated healthy Kinh Vietnamese individuals from southern Vietnam. A total of 28 HLA-A, 41 HLA-B, 21 HLA-C, 26 HLA-DRB1, and 25 HLA-DQB1 alleles were identified. The most frequently occurring HLA alleles were A^∗^11:01:01, B^∗^15:02:01, C^∗^07:02:01, DRB1^∗^12:02:01, and DQB1^∗^03:01:01. Haplotype calculation showed that A^∗^29:01:01∼B^∗^07:05:01, DRB1^∗^12:02:01∼DQB1^∗^3:01:01, A^∗^29:01:01∼C^∗^15:05:02∼B^∗^07:05:01, A^∗^33:03:01∼B^∗^58:01:01∼DRB1^∗^03:01:01, and A^∗^29:01:01∼C^∗^15:05:02∼B^∗^07:05:01∼DRB1^∗^10:01:01∼DQB1^∗^05:01:01 were the most common haplotypes in the southern Kinh Vietnamese population. Allele distribution and haplotype analyses demonstrated that the Vietnamese population shares HLA features with South-East Asians but retains unique characteristics. Data from this study will be potentially applicable in medicine and anthropology.

## Introduction

Human leukocyte antigen (HLA) genes, which encode major histocompatibility complex proteins in humans, are located in the short arm of chromosome 6 (Alper et al., 2006). These encoded HLA proteins are displayed on the cell surface and can be classified into two distinct classes. Class I HLA proteins (A, B, and C) present intracellular antigens originating from viruses or tumors to cytotoxic T lymphocytes. Class II HLA proteins (DR, DQ, and DP) present extracellular antigens to T-helper cells. HLA genes are highly polymorphic and play an important role in immune-mediated diseases, tumor-development processes, transplanted organ or tissue survival determination, and drug hypersensitivity (Dawson et al., 2001; Dhaliwal et al., 2003; Hung et al., 2005; Avila-Rios et al., 2009; Chen et al., 2015; Thao et al., 2018).

HLA genotyping is a complex procedure due to the extreme degree of polymorphism in the major histocompatibility complex family. The most polymorphic regions, known as the core exons, are exons 2 and 3 in HLA class I genes and exon 2 in HLA class II genes. The sequences of the core exons are the most popular targets for genotyping as they are believed to be essential determinants of antigen specificity, which is informative for transplantation. However, in population genetic and evolutionary studies, many polymorphisms in other exons, introns, and UTRs have been identified and contribute to creating HLA nomenclature (Marsh and WHO Nomenclature Committee for Factors of the Hla System, 2012). Currently, HLA typing is performed using DNA-based methods, including SSP- (sequence-specific primer), SSO- (sequence-specific oligonucleotide), and RFLP-PCR (restriction fragment length polymorphism polymerase chain reaction) and sequence-based typing (SBT) (Tait et al., 2009; Bontadini, 2012; Erlich, 2012). SBT was considered the gold-standard method for high-resolution HLA genotyping, although this technique may produce uncertain results due to insufficient sequencing and ambiguous haplotype phasing (Erlich, 2012). Recent advancements in next-generation sequencing (NGS) technologies have significantly impacted the HLA-typing process (Abbott et al., 2006; Bentley et al., 2009; Erlich et al., 2011; Erlich, 2012; Shiina et al., 2012; Hosomichi et al., 2013, 2015; Schöfl et al., 2017). These new approaches can overcome the usual phase ambiguity of HLA alleles and enable massive, parallel, high-resolution HLA-typing. Different NGS-based HLA-typing methods have been established, such as amplicon-based HLA sequencing (Boegel et al., 2012; Shiina et al., 2012; Hosomichi et al., 2013; Schöfl et al., 2017), target enrichment of HLA genes (Wittig et al., 2015), and whole exome or genome sequencing data-derived typing (Liu et al., 2012; Major et al., 2013).

Only a few studies have been performed to analyze HLA allele and haplotype frequency in the Vietnamese population (Vu-Trieu et al., 1997; Busson et al., 2002; Hoa et al., 2008). Moreover, these studies failed to present detailed HLA information due to low-resolution or incomplete loci description. There is an urgent need for an HLA-typing procedure that can yield accurate and detailed HLA allele distribution. Previous studies have investigated HLA allele distribution among the Kinh population in northern Vietnam, but this study aimed to perform high-resolution HLA typing (3-field) via NGS and determine the frequency of specific alleles and haplotypes of HLA-A, -B, -C, -DRB1, and -DQB1 in southern Kinh Vietnamese populations.

## Materials and Methods

### Subjects

A descriptive, cross-sectional study was conducted involving 101 unrelated healthy individuals. All subjects, who originated from Ho Chi Minh City and the surrounding Mekong delta provinces, were self-identified as Kinh Vietnamese and were recruited at the University of Medicine and Pharmacy, Ho Chi Minh City, Vietnam from August to October 2017. The study was approved by the Ethics Committee of the University of Medicine and Pharmacy at Ho Chi Minh City, Vietnam. All subjects were counseled and provided written informed consent for the study.

### DNA Extraction

Venous blood (2 ml) was collected from each subject using an EDTA anticoagulant tube. Genomic DNA was extracted from peripheral blood leukocytes using the QIAamp DNA Mini Kit (Qiagen, Hilden, Germany) according to the manufacturer’s protocol, and samples were stored at −20°C until analysis.

Genomic DNA quality was assessed by measuring absorbance at 260 nm using a NanoDrop 2000 (Thermo Scientific, MA, United States), and the optical density (OD) ratio (260/280 nm) was calculated to evaluate sample purity. The recommended purified genomic DNA concentration (≥30 μg/μL) and OD ratio (≥1.8) for library preparation were ascertained.

### Library Preparation

The HLA TruSight kit (CareDx, Brisbane, CA, United States) was used for library preparation. Library construction began with a long-range PCR for full-length HLA-A, -B, -C, -DRB1, -DQB1 loci. All amplicons were normalized to prevent sequencing bias between samples by using magnetic beads consisting of carboxy-coated paramagnetic particles (Hawkins et al., 1994). The beads bound saturating amounts of DNA, and the DNA concentration was normalized to a similar concentration across samples after the washing and elution steps (Hosomichi et al., 2014). Subsequently, the DNA amplicons were fragmented into approximately 2-kb pieces, indexed, and pooled for sequencing on the MiniSeq platform (Illumina, San Diego, CA, United States). The pooled library was quantitated before loading on MiniSeq as the library concentration determines cluster density, which is an important parameter for data quality. As instructed in the Illumina protocol, a Qubit 3.0 fluorometer (Thermo Scientific, Waltham, MA, United States) was used for library quantitation. The pooled library was loaded unto the MiniSeq system when its concentration was ≥10 ng/μL.

### Sequencing

Next-generation sequencing was performed via the MiniSeq system. Each sample was examined for average depth of coverage and Q30 quality scores, which were >200 and 85, respectively, for all five loci. The sequences were subsequently analyzed using an Assign TruSight HLA v2.0 (CareDx, Brisbane, CA, United States).

### HLA Assigned by Assign TruSight HLA v2.0

Qualified FASTQ files from the MiniSeq system were analyzed by Assign TruSight HLA v2.0 (CareDx, Brisbane, CA, United States). Results with 0 core exon mismatch and phasing ≤2 were accepted. Although full-length HLA loci were sequenced, the maximum resolution that the software Assign TruSight HLA v2.0 can provide is 3-field. Higher resolution (4-field) can be achieved if other analysis tools are applied to assign HLA alleles.

### Statistical Analysis

For single-locus analysis, allele frequencies were calculated by direct counting, deviation from Hardy–Weinberg (HW) proportions was calculated via chi-square test, and the Ewens–Watterson (EW) homozygosity test of neutrality was also performed via Monte-Carlo implementation of the exact test (Ewens, 1972; Watterson, 1978; Slatkin, 1996). The calculation was executed in PyPop: Python for Population Genomics (Lancaster et al., 2007). For multiple-locus analysis, haplotype frequencies were estimated using an expectation-maximization algorithm by Arlequin ver. 3.5 with default settings (Excoffier and Lischer, 2010); linkage disequilibrium (LD) between all HLA allele pairs was analyzed in PyPop, in which D′ and Wn of specific allele pairs were calculated (Lancaster et al., 2007). LD between all HLA loci pairs was further calculated and plotted using conditional asymmetric linkage disequilibrium (ALD) measures (Thomson and Single, 2014). The principal component analysis (PCA) of HLA-A, -B, and -DRB1 was performed using Excel 2010 to compare allele distribution between our data (*n* = 101) and HLA allele frequency data of the Vietnamese Hanoi Kinh population 2 (*n* = 170), Chinese Canton Han population (*n* = 264), Indonesian Sundanese and Javanese population (*n* = 201), Thai population (*n* = 142), Japanese population 3 (*n* = 1018), South Korean population 3 (*n* = 485), and Malaysian Peninsular Malay population (*n* = 951), which were retrieved from the Allele Frequencies Net Database (allelefrequencies.net) (González-Galarza et al., 2015). Due to the unavailability of 3-field HLA data in previous studies, we converted 3-field to 2-field data. For example, HLA-A^∗^24:02:01, A^∗^24:02:13, and A^∗^24:02:40 were converted to HLA-A^∗^24:02 with a frequency (0.13861) that was the sum of the three 3-field alleles (0.12871, 0.00495, and 0.00495, respectively). PCA results were plotted using BioVinci software (BioTuring Inc., San Diego, CA, United States).

## Results

Advancements in NGS offer the ability to distinguish between a set of alleles that share two field names and differ in the third field, such as A^∗^24:02, C^∗^07:01, and DQB1^∗^05:02, in one sequencing batch. As the polymorphisms of A^∗^24:02:40, A^∗^24:02:13, C^∗^07:01:02, and DQB1^∗^05:02:02 are not in the core exons, several traditional PCR and sequencing reactions were required to determine these alleles before NGS methods became available.

### Allele Frequencies

The number of HLA-A, HLA-B, HLA-C, HLA-DRB1, and HLA-DQB1 alleles detected in this study were 28, 41, 21, 26, and 25, respectively. The frequencies of HLA class I and class II alleles are summarized in Table 1. HLA-A^∗^11:01:01, A^∗^24:02:01, and A^∗^33:03:01 (22.77, 12.87, and 10.89%) were the three most frequent HLA-A alleles, followed by A^∗^02:07:01, A^∗^29:01:01, and A^∗^02:03:01 (9.90, 8.42, and 7.43%, respectively). HLA-B^∗^15:02:01, B^∗^46:01:01, B^∗^58:01:01, B^∗^40:01:02, B^∗^38:02:01, and B^∗^07:05:01 (11.88, 9.41, 8.42, 7.92, 7.92, and 6.93%, respectively) were the most frequent HLA-B alleles. The most frequent alleles in locus C were HLA-C^∗^07:02:01, C^∗^01:02:01, and C^∗^08:01:01 (21.78, 13.37, and 12.87%). HLA-DRB1^∗^12:02:01 accounted for 22.28% of the HLA-DRB1 alleles. HLA-DRB1^∗^09:01:02 was the second most frequent allele (13.37%), followed by DRB1^∗^15:02:01, DRB1^∗^10:01:01, DRB1^∗^03:01:01, and DRB1^∗^04:05:01 (9.90, 7.92, 7.42, 6.44%, respectively). On the HLA-DQB1 locus, DQB1^∗^03:01:01 was the most frequent allele (28.71%), followed by DQB1^∗^03:03:02, DQB1^∗^05:01:01, and DQB1^∗^05:02:01 (12.87, 10.89, and 9.90%, respectively).

**TABLE 1:** HLA frequency in the Kinh population (*n* = 101) (AF: allele frequency).

**A**	**Count**	**AF**	**C**	**Count**	**AF**	**B**	**Count**	**AF**	**DRB1**	**Count**	**AF**	**DQB1**	**Count**	**AF**
01:01:01	3	0.01485	01:02:01	27	0.13366	07:02:01	4	0.01980	03:01:01	15	0.07426	02:01:01	14	0.06931
02:01:01	6	0.02970	03:02:02	18	0.08911	07:05:01	14	0.06931	04:01:01	1	0.00495	02:02:01	4	0.01980
02:03:01	15	0.07426	03:03:01	9	0.04455	08:01:01	1	0.00495	04:03:01	3	0.01485	03:01:01	58	0.28713
02:03:02	1	0.00495	03:04:01	15	0.07426	13:01:01	6	0.02970	04:05:01	13	0.06436	03:02:01	5	0.02475
02:06:01	6	0.02970	03:04:02	1	0.00495	13:02:01	2	0.00990	04:06:01	2	0.00990	03:03:02	26	0.12871
02:07:01	20	0.09901	03:17	1	0.00495	15:01:01	2	0.00990	07:01:01	6	0.02970	03:03:05	1	0.00495
03:01:01	1	0.00495	04:01:01	10	0.04950	15:02:01	24	0.11881	08:03:02	11	0.05445	03:05:02	1	0.00495
03:02:01	2	0.00990	04:03:01	15	0.07426	15:11:01	1	0.00495	08:12	1	0.00495	04:01:01	10	0.04950
11:01:01	46	0.22772	04:82	1	0.00495	15:12	3	0.01485	09:01:02	27	0.13366	04:02:01	2	0.00990
11:02:01	5	0.02475	06:02:01	4	0.01980	15:13:01	1	0.00495	10:01:01	16	0.07921	05:01:01	22	0.10891
11:04	2	0.00990	07:01:01	1	0.00495	15:17:01	1	0.00495	11:01:01	5	0.02475	05:01:03	2	0.00990
24:02:01	26	0.12871	07:01:02	1	0.00495	15:25:01	11	0.05446	11:06:01	3	0.01485	05:01:12	1	0.00495
24:02:13	1	0.00495	07:02:01	44	0.21782	15:27:01	1	0.00495	11:129	1	0.00495	05:02:01	20	0.09901
24:02:40	1	0.00495	07:04:01	2	0.00990	15:35	2	0.00990	12:02:01	45	0.22277	05:02:02	2	0.00990
24:03:01	2	0.00990	07:06	1	0.00495	18:01:01	2	0.00990	13:01:01	1	0.00495	05:02:04	1	0.00495
24:07:01	6	0.02970	08:01:01	26	0.12871	18:02	1	0.00495	13:02:01	3	0.01485	05:03:01	5	0.02475
24:10:01	1	0.00495	08:03:01	2	0.00990	27:06	4	0.01980	13:12:01	6	0.02970	05:03:02	1	0.00495
24:20	3	0.01485	12:02:02	3	0.01485	35:01:01	4	0.01980	14:04:01	1	0.00495	05:03:11	1	0.00495
26:01:01	4	0.01980	14:02:01	4	0.01980	35:03:01	1	0.00495	14:05:01	2	0.00990	05:10	1	0.00495
29:01:01	17	0.08416	15:02:01	3	0.01485	35:05:01	7	0.03465	14:10	1	0.00495	05:18	2	0.00990
30:01:01	1	0.00495	15:05:02	14	0.06931	37:01:01	1	0.00495	14:18	1	0.00495	06:01:01	17	0.08416
31:01:02	3	0.01485				38:02:01	16	0.07921	14:54:01	3	0.01485	06:02:01	2	0.00990
32:01:01	1	0.00495	Total	202	1.00000	39:01:01	4	0.01980	15:01:01	5	0.02475	06:03:01	1	0.00495
33:01:01	2	0.00990				39:09:01	1	0.00495	15:02:01	20	0.09901	06:04:01	1	0.00495
33:03:01	22	0.10891				40:01:02	16	0.07921	15:02:02	1	0.00495	06:09:01	2	0.00990
34:01:01	3	0.01485				40:02:01	1	0.00495	16:02:01	9	0.04455			
68:01:02	1	0.00495				40:06:01	4	0.01980				Total	202	1.00000
74:02:01	1	0.00495				44:03:02	2	0.00990	Total	202	1.00000			
						46:01:01	19	0.09406						
Total	202	1.0000				48:01:01	3	0.01485						
						51:01:01	4	0.01980						
						51:02:01	3	0.01485						
						51:06:01	1	0.00495						
						52:01:01	4	0.01980						
						54:01:01	3	0.01485						
						55:02:01	4	0.01980						
						55:18	1	0.00495						
						56:01:01	3	0.01485						
						56:04	2	0.00990						
						57:01:01	1	0.00495						
						58:01:01	17	0.08416						
						Total	202	1.00000						

No tested loci showed any significant departure from the Hardy–Weinberg equilibrium; *p*-values for all homozygotes and all heterozygotes tests were 0.79 & 0.93, 0.73 & 0.93, 0.33 & 0.73, 0.68 & 0.89, and 0.40 & 0.74 for HLA- A, -B, -C, -DRB1, and -DQB1 loci, respectively. The results of the EW homozygosity test of neutrality are summarized in Table 2. *p*-values of F were 0.64, 0.37, 0.22, 0.44, and 0.76 for HLA- A, -B, -C, -DRB1, and -DQB1 loci, respectively.

**TABLE 2:** Results of the Ewens–Watterson homozygosity test of neutrality.

**Locus**	**Number of alleles**	***F*_*obs*_**	***F*_*exp*_**	***p*-value**
A	28	0.1078	0.1055	0.6404
B	41	0.0576	0.0650	0.3709
C	21	0.1117	0.1483	0.2166
DRB1	26	0.1024	0.1154	0.4389
DQB1	25	0.1374	0.1210	0.7593

### Haplotype Frequencies

Tables 3, 4, and 5 list the 20 most common two-locus, three-locus, and five-locus haplotypes. The most frequent haplotypes in the two-locus sets were A^∗^29:01:01∼B^∗^07:05:01 (6.93%), A^∗^33:03:01∼B^∗^58:01:01 (6.43%), A^∗^11:01:01∼B^∗^15:02:01 (5.87%), and DRB1^∗^12:02:01 ∼DQB1^∗^03:01:01 (21.28%), DRB1^∗^09:01:02∼DQB1^∗^03:03:02 (11.88%), DRB1^∗^10:01:01∼DQB1^∗^05:01:01 (7.42%). The two most frequent haplotypes in each three-locus set were A^∗^29:01:01 ∼C^∗^15:05:02∼B^∗^07:05:01 (6.93%) and A^∗^33:03:01∼B^∗^58:01:01 ∼DRB1^∗^03:01:01 (4.95%). The three most frequent five-locus haplotypes were A^∗^29:01:01∼C^∗^15:05:02∼B^∗^07:05:01∼DRB1^∗^ 10:01:01∼DQB1^∗^05:01:01 (4.46%), A^∗^33:03:01∼C^∗^03:02:02 ∼B^∗^58:01:01∼DRB1^∗^03:01:01∼DQB1^∗^02:01:01 (4.46%), and A^∗^11:01:01∼C^∗^08:01:01∼B^∗^15:02:01∼DRB1^∗^12:02:01∼DQB1^∗^ 03:01:01 (3.84%). The likelihood ratio test of linkage disequilibrium demonstrated that all two-, three- and five-locus associations were statistically significant (*p* < 0.001). Data on the full two-locus, three-locus, five-locus, and ten-locus haplotype frequencies are described in Supplementary Tables 1, 2, 3, and 4.

**TABLE 3:** Haplotype frequencies of two-locus HLA.

**A**	**B**	**Est. count**	**hap.freq**	**DRB1**	**DQB1**	**Est. count**	**hap.freq**
29:01:01	07:05:01	14.00	0.06931	12:02:01	03:01:01	43.00	0.21283
33:03:01	58:01:01	13.00	0.06436	09:01:02	03:03:02	24.00	0.11881
11:01:01	15:02:01	11.86	0.05869	10:01:01	05:01:01	15.00	0.07426
02:07:01	46:01:01	10.40	0.05146	03:01:01	02:01:01	14.00	0.06931
02:03:01	38:02:01	7.92	0.03922	08:03:02	06:01:01	11.00	0.05446
11:01:01	40:01:02	7.70	0.03810	04:05:01	04:01:01	10.00	0.04950
11:01:01	38:02:01	4.69	0.02322	16:02:01	05:02:01	9.00	0.04455
02:07:01	15:02:01	4.15	0.02052	13:12:01	03:01:01	6.00	0.02970
24:02:01	27:06:00	4.00	0.01980	15:02:01	05:01:01	6.00	0.02970
24:07:01	35:05:01	4.00	0.01980	15:02:01	05:02:01	5.99	0.02966
11:01:01	13:01:01	3.82	0.01891	07:01:01	02:02:01	4.00	0.01980
11:01:01	15:25:01	3.61	0.01789	11:01:01	03:01:01	4.00	0.01980
24:02:01	46:01:01	3.28	0.01624	04:03:01	03:02:01	3.00	0.01485
11:01:01	39:01:01	3.00	0.01485	04:05:01	04:02:01	2.00	0.00990
24:02:01	15:02:01	3.00	0.01485	04:06:01	03:02:01	2.00	0.00990
24:02:01	15:25:01	2.39	0.01181	11:06:01	03:01:01	2.00	0.00990
11:01:01	46:01:01	2.33	0.01151	13:02:01	06:09:01	2.00	0.00990
24:02:01	40:01:02	2.23	0.01102	14:05:01	05:03:01	2.00	0.00990
02:01:01	35:01:01	2.00	0.00990	14:54:01	05:02:01	2.00	0.00990
02:01:01	40:01:02	2.00	0.00990	15:01:01	06:01:01	2.00	0.00990

**TABLE 4:** Haplotype frequencies of three-locus HLA.

**A**	**C**	**B**	**Est. count**	**hap.freq**	**A**	**B**	**DRB1**	**Est. count**	**hap.freq**
29:01:01	15:05:02	07:05:01	14.00	0.06931	33:03:01	58:01:01	03:01:01	10.00	0.04950
33:03:01	03:02:02	58:01:01	13.00	0.06436	29:01:01	07:05:01	10:01:01	9.00	0.04455
11:01:01	08:01:01	15:02:01	10.84	0.05367	11:01:01	15:02:01	12:02:01	8.43	0.04175
02:07:01	01:02:01	46:01:01	10.38	0.05138	02:07:01	46:01:01	09:01:02	5.83	0.02886
02:03:01	07:02:01	38:02:01	8.00	0.03960	02:03:01	38:02:01	08:03:02	4.00	0.01980
11:01:01	04:03:01	15:25:01	5.00	0.02475	11:01:01	40:01:02	12:02:01	4.00	0.01980
11:01:01	07:02:01	38:02:01	5.00	0.02475	24:02:01	27:06:00	12:02:01	4.00	0.01980
11:01:01	07:02:01	40:01:02	5.00	0.02475	29:01:01	07:05:01	09:01:02	4.00	0.01980
02:07:01	08:01:01	15:02:01	4.16	0.02059	02:07:01	15:02:01	12:02:01	3.17	0.01569
24:07:01	04:01:01	35:05:01	4.00	0.01980	11:01:01	46:01:01	09:01:02	3.17	0.01569
11:01:01	03:04:01	13:01:01	3.82	0.01891	24:02:01	15:25:01	15:02:01	3.00	0.01485
24:02:01	01:02:01	46:01:01	3.28	0.01625	24:02:01	35:05:01	08:03:02	3.00	0.01485
24:02:01	04:03:01	15:25:01	3.00	0.01485	24:02:01	15:02:01	12:02:01	2.40	0.01187
24:02:01	03:04:01	27:06:00	3.00	0.01485	02:01:01	35:01:01	07:01:01	2.00	0.00990
11:01:01	01:02:01	46:01:01	2.34	0.01158	02:01:01	40:01:02	09:01:02	2.00	0.00990
02:01:01	03:03:01	35:01:01	2.00	0.00990	02:03:01	38:02:01	16:02:01	2.00	0.00990
11:01:01	07:02:01	39:01:01	2.00	0.00990	02:07:01	46:01:01	11:01:01	2.00	0.00990
11:02:01	07:02:01	40:01:02	2.00	0.00990	02:07:01	46:01:01	12:02:01	2.00	0.00990
24:02:01	08:01:01	15:02:01	2.00	0.00990	11:01:01	07:02:01	10:01:01	2.00	0.00990
24:02:01	04:01:01	35:05:01	2.00	0.00990	11:01:01	15:02:01	07:01:01	2.00	0.00990

**TABLE 5:** Haplotype frequencies of five-locus HLA.

**A**	**C**	**B**	**DRB1**	**DQB1**	**Est. count**	**hap.freq**
29:01:01	15:05:02	07:05:01	10:01:01	05:01:01	9.00	0.04455
33:03:01	03:02:02	58:01:01	03:01:01	02:01:01	9.00	0.04455
11:01:01	08:01:01	15:02:01	12:02:01	03:01:01	7.76	0.03844
02:07:01	01:02:01	46:01:01	09:01:02	03:03:02	5.76	0.02854
11:01:01	01:02:01	46:01:01	09:01:02	03:03:02	4.23	0.02096
02:03:01	07:02:01	38:02:01	08:03:02	06:01:01	4.00	0.01980
02:07:01	08:01:01	15:02:01	12:02:01	03:01:01	3.23	0.01601
11:01:01	07:02:01	40:01:02	12:02:01	03:01:01	3.00	0.01485
24:07:01	04:01:01	35:05:01	12:02:01	03:01:01	3.00	0.01485
29:01:01	15:05:02	07:05:01	09:01:02	03:03:02	3.00	0.01485
02:01:01	03:03:01	35:01:01	07:01:01	02:02:01	2.00	0.00990
02:03:01	07:02:01	38:02:01	12:02:01	05:02:01	2.00	0.00990
02:07:01	08:01:01	15:02:01	13:12:01	03:01:01	2.00	0.00990
02:07:01	01:02:01	46:01:01	12:02:01	03:01:01	2.00	0.00990
11:01:01	07:02:01	07:02:01	10:01:01	05:01:01	2.00	0.00990
11:01:01	08:01:01	15:02:01	07:01:01	02:02:01	2.00	0.00990
11:01:01	07:02:01	15:25:01	15:02:01	03:01:01	2.00	0.00990
11:01:01	07:02:01	38:02:01	15:02:01	05:01:01	2.00	0.00990
11:01:01	07:02:01	39:01:01	14:54:01	03:01:01	2.00	0.00990
11:01:01	03:02:02	58:01:01	03:01:01	02:01:01	2.00	0.00990

### Population Genetic Analysis

**FIGURE 1:**
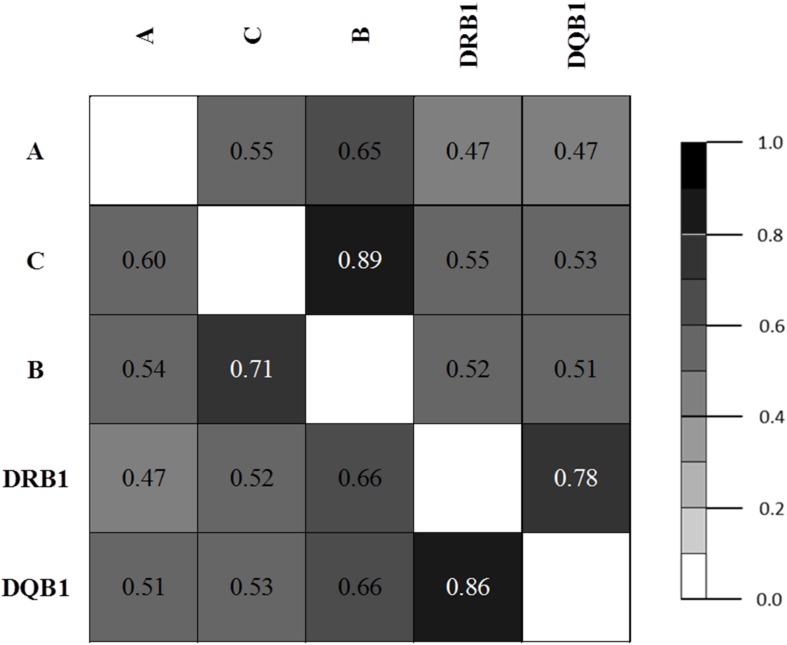
LD plot based on asymmetric linkage disequilibrium (ALD) measures for HLA genes.

Pairwise LD estimates are given in Table 6 with D′ and Wn. The LD of allele pairs was always statistically significant with 1,000 permutations. LD plots based on ALD measures for HLA loci are shown in Figure 1. Generally, the associations between HLA loci within HLA classes were stronger than between HLA loci in different classes, except for the case of B & DRB1 loci. Both symmetric and asymmetric LD showed that the strongest genetic linkages were between C & B loci and DRB1 & DQB1 loci.

**TABLE 6:** Pairwise linkage disequilibrium estimates.

**Locus pair**	**D′**	**Wn**	**#permutations**	***p*-value**
A:C	0.68522	0.56686	999	0.0000
A:B	0.76923	0.61971	999	0.0000
A:DRB1	0.63441	0.51613	999	0.0000
A:DQB1	0.63335	0.48973	999	0.0000
C:B	0.91995	0.84507	999	0.0000
C:DRB1	0.66870	0.51054	999	0.0000
C:DQB1	0.65889	0.48280	999	0.0000
B:DRB1	0.79814	0.58696	999	0.0000
B:DQB1	0.77352	0.61127	999	0.0000
DRB1:DQB1	0.93176	0.70996	999	0.0000

**FIGURE 2:**
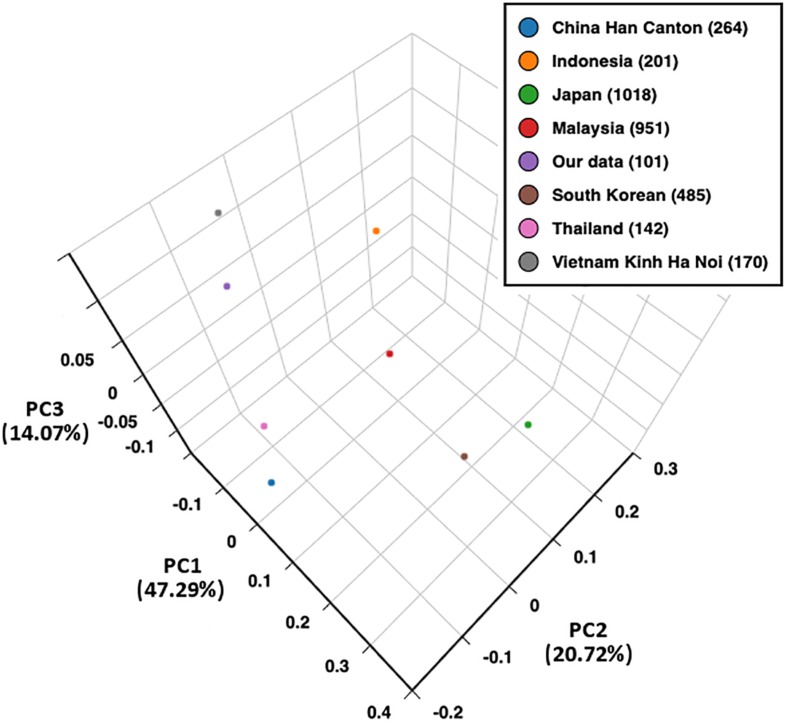
Principal component analysis (PCA) plot of eight populations based on HLA-A, -B, and -DRB1 allele frequencies. PC1, principal component 1; PC2, principal component 2; PC3, principal component 3.

The PCA plot of eight Asian populations is shown in Figure 2. The percentage of variability represented by the first three principal components was 82.08%. The first, second, and third principal components demonstrated 47.29, 20.72, and 14.07% of the variances in allele frequencies between populations, respectively. The first principal component distinguished between the South-East Asian, Han Chinese, and East Asian (Japanese and South Korean) populations. The second principal component separated the Han Chinese, Kinh Vietnamese, and Thai from the Indonesian and Malaysian populations. The third principal component distinguished the Kinh Vietnamese from the Han Chinese and other South-East Asian populations. A homogeneous allele frequency distribution of HLA-A, -B, and -DRB1 was observed between the northern and southern Kinh Vietnamese (Hoa et al., 2008). Japanese and South Korean also presented a similar distribution of HLA alleles.

## Discussion

In recent years, various HLA-typing methods using different NGS approaches have been performed. NGS-based HLA typing can provide high-resolution, unambiguous, phase-defined HLA alleles, avoiding several limitations compared to traditional sequence-based typing methods (Carapito et al., 2016). Our study showed the distribution of HLA-A, -B, -C, -DRB1, and -DQB1 alleles and haplotypes among the southern Kinh Vietnamese population using high-resolution NGS typing (reported at 3-field resolution, which remains ambiguous in many cases). Highly polymorphic sequences at both HLA class I and class II loci resulted in 28 alleles for HLA-A, 41 alleles for HLA-B, 21 alleles for HLA-C, 26 alleles for HLA-DRB1, and 25 alleles for HLA-DQB1.

The most frequent HLA-A alleles found in this study were A^∗^11:01:01 and A^∗^24:02:01. The high frequency of HLA-A^∗^11:01 and A^∗^24:02:01 is consistent with previous typing results of northern Kinh Vietnamese and other Asian populations, such as the Chinese, Thai, Indonesian, Korean, and Japanese (Lee et al., 2005; Hoa et al., 2008; Yuliwulandari et al., 2009; Shen et al., 2014; Ikeda et al., 2015; Nakkam et al., 2018). Among HLA-C alleles identified in this study, C^∗^07:02:01 was found to be widely distributed globally, while C^∗^01:02:01 was common in Asians (Lee et al., 2005; Shen et al., 2014; Ikeda et al., 2015; Nakkam et al., 2018). The predominance of HLA-B^∗^15 alleles is a major distinguishing characteristic of the Kinh population from the Thai and Chinese groups (Shen et al., 2014; Nakkam et al., 2018). However, this predominance is similar in the Indonesian population (Yuliwulandari et al., 2009). Detailed comparison of B^∗^15 alleles among the Vietnamese and Indonesians showed similar popularity of B^∗^15:02, while the second most-frequent B^∗^15 alleles were B^∗^15:25:01 and B^∗^15:13, respectively. HLA-B^∗^07:05:01, the only B^∗^07 allele found in Kinh Vietnamese, was the sixth most-frequent HLA-B allele, whereas it is a minor allele in other Asian groups (Whang et al., 2001).

At the HLA-DRB1 locus, the most frequent allele was HLA-DRB1^∗^12:02:01 (22.28%), which is common among South-East Asian populations (Busson et al., 2002; Hoa et al., 2008; Yuliwulandari et al., 2009; Nakkam et al., 2018) but infrequent among Northern East Asian groups, including Japanese and Koreans (Lee et al., 2005; Ikeda et al., 2015). Another similarity observed between the Kinh Vietnamese, Muong Vietnamese, and other South-East Asians is the predominance of HLA-DRB1^∗^15:02:01 over HLA-DRB1^∗^15:01:01, in contrast to what was observed among Northern East Asian populations. The first and second-most predominance of HLA-DQB1^∗^03:01:01 (28.71%) and DQB1^∗^03:03:02 (12.38%) in Kinh Vietnamese is similar among East Asian populations, including Taiwanese, Chinese, Korean, and Japanese (Saito et al., 2000; Lee et al., 2005; Yang and Chen, 2017), while the third-most predominance of HLA-DQB1^∗^05:02:01 (9.90%) is closer to the characteristics of the Thai population (Romphruk et al., 1999). In Kinh Vietnamese, the predominance of DQB1^∗^05:01 over DQB1^∗^05:02 in our data was consistent with data from a previous study (Hoa et al., 2008). However, Muong Vietnamese showed a contrary distribution (48%) of DQB1^∗^05:02 (Busson et al., 2002).

Based on the haplotype calculation, most two-, three-, and five-locus HLA haplotypes with predominant frequencies were consistent with a previous report on northern Kinh Vietnamese (Hoa et al., 2008). Despite being the sixth most common HLA-B allele, B^∗^07:05:01 was strongly associated with A^∗^29:01:01 and lead to the common signature haplotypes of the Kinh population, including A^∗^29:01:01∼B^∗^07:05:01, A^∗^29:01:01∼C^∗^15:05:02∼B^∗^07:05:01, and A^∗^29:01:01∼B^∗^07:05:01∼DRB1^∗^10:01:01. Interestingly, A^∗^29:01:01∼C^∗^15:05:02∼B^∗^07:05:01∼DRB1^∗^10:01:01∼DQB1^∗^ 05:01:01 was the most common five-locus haplotype (4.45%). The predominance of these haplotypes might be a unique feature of the Kinh Vietnamese. The strong association of DRB1^∗^12:02:01 and DQB1^∗^03:01:01 in HLA class II found in our study is also well-described in Thai, Indonesian, and surrounding populations (Gao et al., 1992; Romphruk et al., 1999; Mack et al., 2000).

The strong associations between all pairs of HLA loci in southern Kinh Vietnamese indicate a low probability of recombination between alleles from these loci; therefore, individuals who carry allele haplotypes in LD are more likely to find a donor with matching haplotypes. The strong LD between class I HLA loci has also been well-described in Asian populations (Shen et al., 2014; Ikeda et al., 2015), while the nearly complete LD of DRB1 and DQB1 loci has been observed in Han Chinese (Trachtenberg et al., 2007). PCA showed a homogeneous HLA-A, -B, and -DRB1 allele distribution of northern and southern Kinh Vietnamese. The allele distribution also demonstrated a closer relationship between Kinh Vietnamese and other South-East Asian groups than with the Han Chinese group. The Japanese were closely grouped with South Koreans, reflecting the similarity in HLA distribution among East Asian populations.

Previously, HLA typing of Asian populations were mainly based on SSO-PCR (Lee et al., 2005; Yuliwulandari et al., 2009; Shen et al., 2014; Ikeda et al., 2015; Nakkam et al., 2018). Due to the finite amounts of probes designed to recognize the polymorphisms in the core exons, this technique only allows certain allele typing with 2-field resolution. Alleles were then assigned by software based on SSO-PCR patterns. Hence, the number of alleles determined by SSO-PCR is limited. With full-length HLA sequences provided by NGS, HLA-typing software programs align sequence reads to the entire IMGT/HLA Database to find the best-matching alleles. NGS-based typing, therefore, can provide diversified HLA assignments. In our study, the number of identified alleles (141 alleles) in 101 subjects was higher compared to the previous study in northern Kinh Vietnamese (115 identified alleles in 170 subjects) (Hoa et al., 2008). Similar results were obtained in the Thai population, in which the number of HLA alleles determined by NGS and SSO-PCR were 156 and 144, respectively (Geretz et al., 2018; Nakkam et al., 2018).

Recently, it has been shown that both high-resolution HLA typing and haplotyping are important in hematopoietic stem cell transplantation for both unrelated and related donors in reducing post-transplantation adverse outcomes (Agarwal et al., 2017; Buhler et al., 2019); a single high-resolution HLA mismatch may lead to a similar negative effect on outcomes as a low-resolution one (Fuji et al., 2015; Armstrong et al., 2017). Therefore, it has been suggested that high-resolution HLA typing can reduce the likelihood of missing a clinically significant mismatch compared to traditional low-resolution typing, especially in developing countries where high-resolution HLA typing methods are not widely available (Agarwal et al., 2017). With a 3-field resolution, our typing process can distinguish between HLA-A^∗^24:02:01, HLA-A^∗^24:02:13, and HLA-A^∗^24:02:40 and between HLA-C^∗^07:01:01 and HLA-C^∗^07:01:02, which are considered high-resolution mismatches. Although traditional SBT can separate these alleles, it is time and resource-consuming.

Our study had several limitations that should be considered in interpreting the results. First of all, the absence of other class II HLA descriptions (HLA-DQA1, -DPA1, and -DPB1) makes the study less informative, especially for population genetic purposes. Second, the study sample size was relatively small. This may increase the risk of missing rare HLA alleles in Kinh Vietnamese and reduce the significance of statistical analysis. These limitations will necessitate further studies with comprehensive allele descriptions and larger sample sizes.

It is now also well-recognized that HLA molecules are strongly associated with the pathophysiology of adverse drug reactions, including severe cutaneous adverse reaction (SCAR), agranulocytosis, and liver injury. High prevalence of HLA-B^∗^15:02, B^∗^58:01, B^∗^38:02, DRB1^∗^08:03, and C^∗^03:02 suggests that the Kinh Vietnamese population is at a high risk of developing carbamazepine-induced SCAR, allopurinol-induced SCAR, methimazole-induced agranulocytosis, and methimazole-induced liver injury, respectively (Hung et al., 2005; Chen et al., 2015; Thao et al., 2018; Li et al., 2019), while the risk of developing dapsone or abacavir-induced hypersensitivity is low due to the low prevalence of HLA-B^∗^13:01 and B^∗^57:01 (Mallal et al., 2008; Sousa-Pinto et al., 2015; Tempark et al., 2017). Therefore, HLA information is important to clinicians for treatment modality adoption and to healthcare policymakers for constructing personalized medicine strategies.

## Conclusion

To our knowledge, this is the first report of high-resolution HLA-A, -B, -C, -DRB1, and -DQB1 allele and haplotype frequencies in southern Kinh Vietnamese individuals. These data display the homogenous distribution of HLA between the northern and southern Kinh population in Vietnam. Although the characteristics of HLA class I and II alleles and haplotypes in the Kinh Vietnamese are similar to those in the Thai, Malaysian, and Indonesian populations, they still retain unique characteristics. Data from this study will be useful in anthropology, immune-mediated diseases, transplantation therapy, and drug hypersensitivity.

## Data Availability Statement

Raw data supporting the conclusions of this article are available on NCBI SRA with accession PRJNA609593. The data on HLA allele frequencies and haplotypes presented in this study are available on allelefrequencies.net with accession Vietnam Kinh (*n* = 101).

## Ethics Statement

The studies involving human participants were reviewed and approved by The Ethics committee of University of Medicine and Pharmacy at Ho Chi Minh City, Vietnam. The patients/participants provided their written informed consent to participate in this study.

## Author Contributions

TM and MD designed the study, wrote the manuscript. MD, LL, and VN performed the experiments. TD, HV, NN, MD, and TM analyzed the data.

## Conflict of Interest

The authors declare that the research was conducted in the absence of any commercial or financial relationships that could be construed as a potential conflict of interest.
