# Supplementary material for: High-Resolution HLA Typing of HLA-A, -B, -C, -DRB1, and -DQB1 in Kinh Vietnamese by Using Next-Generation Sequencing
Source: Front Genet. 2020 Apr 30;11:383. doi: 10.3389/fgene.2020.00383 (PMC7204072; doi:10.3389/fgene.2020.00383)
Supplement: Supplementary file 2 [file Table_2.DOCX]

Supplementary table 2. Haplotype frequencies of three-locus HLA

| A | C | B | Est. count | hap.freq | A | B | DRB1 | Est. count | hap.freq |
| --- | --- | --- | --- | --- | --- | --- | --- | --- | --- |
| 29:01:01 | 15:05:02 | 07:05:01 | 14.00 | 0.06931 | 33:03:01 | 58:01:01 | 03:01:01 | 10.00 | 0.04950 |
| 33:03:01 | 03:02:02 | 58:01:01 | 13.00 | 0.06436 | 29:01:01 | 07:05:01 | 10:01:01 | 9.00 | 0.04455 |
| 11:01:01 | 08:01:01 | 15:02:01 | 10.84 | 0.05367 | 11:01:01 | 15:02:01 | 12:02:01 | 8.43 | 0.04175 |
| 02:07:01 | 01:02:01 | 46:01:01 | 10.38 | 0.05138 | 02:07:01 | 46:01:01 | 09:01:02 | 5.83 | 0.02886 |
| 02:03:01 | 07:02:01 | 38:02:01 | 8.00 | 0.03960 | 02:03:01 | 38:02:01 | 08:03:02 | 4.00 | 0.01980 |
| 11:01:01 | 04:03:01 | 15:25:01 | 5.00 | 0.02475 | 11:01:01 | 40:01:02 | 12:02:01 | 4.00 | 0.01980 |
| 11:01:01 | 07:02:01 | 38:02:01 | 5.00 | 0.02475 | 24:02:01 | 27:06:00 | 12:02:01 | 4.00 | 0.01980 |
| 11:01:01 | 07:02:01 | 40:01:02 | 5.00 | 0.02475 | 29:01:01 | 07:05:01 | 09:01:02 | 4.00 | 0.01980 |
| 02:07:01 | 08:01:01 | 15:02:01 | 4.16 | 0.02059 | 02:07:01 | 15:02:01 | 12:02:01 | 3.17 | 0.01569 |
| 24:07:01 | 04:01:01 | 35:05:01 | 4.00 | 0.01980 | 11:01:01 | 46:01:01 | 09:01:02 | 3.17 | 0.01569 |
| 11:01:01 | 03:04:01 | 13:01:01 | 3.82 | 0.01891 | 24:02:01 | 15:25:01 | 15:02:01 | 3.00 | 0.01485 |
| 24:02:01 | 01:02:01 | 46:01:01 | 3.28 | 0.01625 | 24:02:01 | 35:05:01 | 08:03:02 | 3.00 | 0.01485 |
| 24:02:01 | 04:03:01 | 15:25:01 | 3.00 | 0.01485 | 24:02:01 | 15:02:01 | 12:02:01 | 2.40 | 0.01187 |
| 24:02:01 | 03:04:01 | 27:06:00 | 3.00 | 0.01485 | 02:01:01 | 35:01:01 | 07:01:01 | 2.00 | 0.00990 |
| 11:01:01 | 01:02:01 | 46:01:01 | 2.34 | 0.01158 | 02:01:01 | 40:01:02 | 09:01:02 | 2.00 | 0.00990 |
| 02:01:01 | 03:03:01 | 35:01:01 | 2.00 | 0.00990 | 02:03:01 | 38:02:01 | 16:02:01 | 2.00 | 0.00990 |
| 11:01:01 | 07:02:01 | 39:01:01 | 2.00 | 0.00990 | 02:07:01 | 46:01:01 | 11:01:01 | 2.00 | 0.00990 |
| 11:02:01 | 07:02:01 | 40:01:02 | 2.00 | 0.00990 | 02:07:01 | 46:01:01 | 12:02:01 | 2.00 | 0.00990 |
| 24:02:01 | 08:01:01 | 15:02:01 | 2.00 | 0.00990 | 11:01:01 | 07:02:01 | 10:01:01 | 2.00 | 0.00990 |
| 24:02:01 | 04:01:01 | 35:05:01 | 2.00 | 0.00990 | 11:01:01 | 15:02:01 | 07:01:01 | 2.00 | 0.00990 |
| 24:02:01 | 03:02:02 | 58:01:01 | 2.00 | 0.00990 | 11:01:01 | 38:02:01 | 15:02:01 | 2.00 | 0.00990 |
| 24:07:01 | 08:01:01 | 15:02:01 | 2.00 | 0.00990 | 11:01:01 | 39:01:01 | 14:54:01 | 2.00 | 0.00990 |
| 24:20:00 | 08:03:01 | 48:01:01 | 2.00 | 0.00990 | 24:02:01 | 13:01:01 | 16:02:01 | 2.00 | 0.00990 |
| 29:01:01 | 08:01:01 | 15:02:01 | 2.00 | 0.00990 | 24:07:01 | 15:02:01 | 15:02:01 | 2.00 | 0.00990 |
| 31:01:02 | 15:02:01 | 51:02:01 | 2.00 | 0.00990 | 24:07:01 | 35:05:01 | 09:01:02 | 2.00 | 0.00990 |
| 33:03:01 | 01:02:01 | 56:04:00 | 2.00 | 0.00990 | 29:01:01 | 15:02:01 | 12:02:01 | 2.00 | 0.00990 |
| 02:07:01 | 14:02:01 | 51:01:01 | 1.46 | 0.00724 | 31:01:02 | 51:02:01 | 08:03:02 | 2.00 | 0.00990 |
| 24:02:01 | 03:04:01 | 13:01:01 | 1.18 | 0.00584 | 34:01:01 | 56:01:01 | 04:05:01 | 2.00 | 0.00990 |
| 01:01:01 | 07:01:02 | 15:17:01 | 1.00 | 0.00495 | 11:01:01 | 58:01:01 | 03:01:01 | 1.40 | 0.00692 |
| 01:01:01 | 06:02:01 | 57:01:01 | 1.00 | 0.00495 | 01:01:01 | 15:17:01 | 13:02:01 | 1.00 | 0.00495 |
| 01:01:01 | 03:02:02 | 58:01:01 | 1.00 | 0.00495 | 01:01:01 | 57:01:01 | 07:01:01 | 1.00 | 0.00495 |
| 02:01:01 | 03:17 | 40:01:02 | 1.00 | 0.00495 | 01:01:01 | 58:01:01 | 13:02:01 | 1.00 | 0.00495 |
| 02:01:01 | 04:03:01 | 40:01:02 | 1.00 | 0.00495 | 02:01:01 | 54:01:01 | 11:01:01 | 1.00 | 0.00495 |
| 02:01:01 | 08:01:01 | 48:01:01 | 1.00 | 0.00495 | 02:01:01 | 55:02:01 | 15:01:01 | 1.00 | 0.00495 |
| 02:01:01 | 04:03:01 | 55:02:01 | 1.00 | 0.00495 | 02:03:01 | 38:02:01 | 04:03:01 | 1.00 | 0.00495 |
| 02:03:01 | 07:02:01 | 39:09:01 | 1.00 | 0.00495 | 02:03:01 | 38:02:01 | 11:01:01 | 1.00 | 0.00495 |
| 02:03:01 | 04:03:01 | 40:01:02 | 1.00 | 0.00495 | 02:03:01 | 39:09:01 | 15:02:01 | 1.00 | 0.00495 |
| 02:03:01 | 04:03:01 | 51:01:01 | 1.00 | 0.00495 | 02:03:01 | 40:01:02 | 12:02:01 | 1.00 | 0.00495 |
| 02:03:01 | 14:02:01 | 51:01:01 | 1.00 | 0.00495 | 02:03:01 | 51:01:01 | 04:05:01 | 1.00 | 0.00495 |
| 02:03:01 | 12:02:02 | 52:01:01 | 1.00 | 0.00495 | 02:03:01 | 51:01:01 | 14:05:01 | 1.00 | 0.00495 |
| 02:03:01 | 01:02:01 | 54:01:01 | 1.00 | 0.00495 | 02:03:01 | 52:01:01 | 15:02:01 | 1.00 | 0.00495 |
| 02:03:01 | 01:02:01 | 55:02:01 | 1.00 | 0.00495 | 02:03:01 | 54:01:01 | 04:05:01 | 1.00 | 0.00495 |
| 02:03:02 | 01:02:01 | 18:01:01 | 1.00 | 0.00495 | 02:03:01 | 55:02:01 | 15:02:01 | 1.00 | 0.00495 |
| 02:06:01 | 07:02:01 | 15:01:01 | 1.00 | 0.00495 | 02:03:02 | 54:01:01 | 14:04:01 | 1.00 | 0.00495 |
| 02:06:01 | 03:02:02 | 15:13:01 | 1.00 | 0.00495 | 02:06:01 | 15:01:01 | 11:06:01 | 1.00 | 0.00495 |
| 02:06:01 | 07:02:01 | 15:25:01 | 1.00 | 0.00495 | 02:06:01 | 15:25:01 | 16:02:01 | 1.00 | 0.00495 |
| 02:06:01 | 03:03:01 | 35:01:01 | 1.00 | 0.00495 | 02:06:01 | 38:02:01 | 04:05:01 | 1.00 | 0.00495 |
| 02:06:01 | 07:02:01 | 38:02:01 | 1.00 | 0.00495 | 02:06:01 | 40:01:02 | 08:03:02 | 1.00 | 0.00495 |
| 02:06:01 | 03:04:01 | 40:01:02 | 1.00 | 0.00495 | 02:06:01 | 40:01:02 | 12:02:01 | 1.00 | 0.00495 |
| 02:07:01 | 07:02:01 | 07:02:01 | 1.00 | 0.00495 | 02:06:01 | 52:01:01 | 09:01:02 | 1.00 | 0.00495 |
| 02:07:01 | 07:02:01 | 08:01:01 | 1.00 | 0.00495 | 02:07:01 | 07:02:01 | 14:54:01 | 1.00 | 0.00495 |
| 02:07:01 | 01:02:01 | 15:12 | 1.00 | 0.00495 | 02:07:01 | 07:05:01 | 13:12:01 | 1.00 | 0.00495 |
| 02:07:01 | 03:04:02 | 27:06:00 | 1.00 | 0.00495 | 02:07:01 | 08:01:01 | 03:01:01 | 1.00 | 0.00495 |
| 03:01:01 | 03:04:01 | 07:02:01 | 1.00 | 0.00495 | 02:07:01 | 15:12 | 12:02:01 | 1.00 | 0.00495 |
| 03:02:01 | 06:02:01 | 13:02:01 | 1.00 | 0.00495 | 02:07:01 | 46:01:01 | 15:02:01 | 1.00 | 0.00495 |
| 03:02:01 | 06:02:01 | 38:02:01 | 1.00 | 0.00495 | 02:07:01 | 51:01:01 | 16:02:01 | 1.00 | 0.00495 |
| 11:01:01 | 07:02:01 | 15:02:01 | 1.00 | 0.00495 | 02:07:01 | 58:01:01 | 03:01:01 | 1.00 | 0.00495 |
| 11:01:01 | 03:03:01 | 15:12 | 1.00 | 0.00495 | 03:01:01 | 40:01:02 | 14:05:01 | 1.00 | 0.00495 |
| 11:01:01 | 07:04:01 | 18:01:01 | 1.00 | 0.00495 | 03:02:01 | 13:02:01 | 04:05:01 | 1.00 | 0.00495 |
| 11:01:01 | 07:01:01 | 37:01:01 | 1.00 | 0.00495 | 03:02:01 | 38:02:01 | 09:01:02 | 1.00 | 0.00495 |
| 11:01:01 | 03:03:01 | 40:01:02 | 1.00 | 0.00495 | 11:01:01 | 15:01:01 | 09:01:02 | 1.00 | 0.00495 |
| 11:01:01 | 03:04:01 | 40:01:02 | 1.00 | 0.00495 | 11:01:01 | 15:02:01 | 13:12:01 | 1.00 | 0.00495 |
| 11:01:01 | 07:02:01 | 40:02:01 | 1.00 | 0.00495 | 11:01:01 | 15:12 | 12:02:01 | 1.00 | 0.00495 |
| 11:01:01 | 03:04:01 | 44:03:02 | 1.00 | 0.00495 | 11:01:01 | 15:25:01 | 10:01:01 | 1.00 | 0.00495 |
| 11:01:01 | 14:02:01 | 51:06:01 | 1.00 | 0.00495 | 11:01:01 | 15:25:01 | 13:12:01 | 1.00 | 0.00495 |
| 11:01:01 | 07:02:01 | 55:02:01 | 1.00 | 0.00495 | 11:01:01 | 15:25:01 | 16:02:01 | 1.00 | 0.00495 |
| 11:01:01 | 07:02:01 | 55:18:00 | 1.00 | 0.00495 | 11:01:01 | 18:01:01 | 11:06:01 | 1.00 | 0.00495 |
| 11:01:01 | 04:03:01 | 56:01:01 | 1.00 | 0.00495 | 11:01:01 | 35:01:01 | 15:01:01 | 1.00 | 0.00495 |
| 11:02:01 | 07:02:01 | 15:25:01 | 1.00 | 0.00495 | 11:01:01 | 37:01:01 | 10:01:01 | 1.00 | 0.00495 |
| 11:02:01 | 07:02:01 | 15:35 | 1.00 | 0.00495 | 11:01:01 | 38:02:01 | 08:12 | 1.00 | 0.00495 |
| 11:02:01 | 07:02:01 | 39:01:01 | 1.00 | 0.00495 | 11:01:01 | 38:02:01 | 11:129 | 1.00 | 0.00495 |
| 11:04 | 01:02:01 | 46:01:01 | 1.00 | 0.00495 | 11:01:01 | 39:01:01 | 15:01:01 | 1.00 | 0.00495 |
| 11:04 | 15:02:01 | 51:02:01 | 1.00 | 0.00495 | 11:01:01 | 40:01:02 | 04:05:01 | 1.00 | 0.00495 |
| 24:02:01 | 03:04:01 | 15:02:01 | 1.00 | 0.00495 | 11:01:01 | 40:01:02 | 11:01:01 | 1.00 | 0.00495 |
| 24:02:01 | 03:03:01 | 35:01:01 | 1.00 | 0.00495 | 11:01:01 | 40:02:01 | 04:05:01 | 1.00 | 0.00495 |
| 24:02:01 | 04:01:01 | 35:03:01 | 1.00 | 0.00495 | 11:01:01 | 44:03:02 | 13:12:01 | 1.00 | 0.00495 |
| 24:02:01 | 03:03:01 | 40:01:02 | 1.00 | 0.00495 | 11:01:01 | 52:01:01 | 14:10 | 1.00 | 0.00495 |
| 24:02:01 | 04:82 | 40:01:02 | 1.00 | 0.00495 | 11:01:01 | 55:02:01 | 09:01:02 | 1.00 | 0.00495 |
| 24:02:01 | 03:04:01 | 40:06:01 | 1.00 | 0.00495 | 11:01:01 | 55:18:00 | 04:05:01 | 1.00 | 0.00495 |
| 24:02:01 | 08:01:01 | 40:06:01 | 1.00 | 0.00495 | 11:01:01 | 56:01:01 | 04:05:01 | 1.00 | 0.00495 |
| 24:02:01 | 07:02:01 | 52:01:01 | 1.00 | 0.00495 | 11:01:01 | 58:01:01 | 16:02:01 | 1.00 | 0.00495 |
| 24:02:01 | 07:02:01 | 54:01:01 | 1.00 | 0.00495 | 11:02:01 | 15:25:01 | 12:02:01 | 1.00 | 0.00495 |
| 24:02:13 | 12:02:02 | 52:01:01 | 1.00 | 0.00495 | 11:02:01 | 15:25:01 | 15:02:01 | 1.00 | 0.00495 |
| 24:02:40 | 04:01:01 | 35:05:01 | 1.00 | 0.00495 | 11:02:01 | 15:35 | 15:02:01 | 1.00 | 0.00495 |
| 24:03:01 | 07:02:01 | 15:35 | 1.00 | 0.00495 | 11:02:01 | 40:01:02 | 08:03:02 | 1.00 | 0.00495 |
| 24:03:01 | 03:03:01 | 44:03:02 | 1.00 | 0.00495 | 11:02:01 | 40:01:02 | 15:01:01 | 1.00 | 0.00495 |
| 24:10:01 | 07:04:01 | 18:02 | 1.00 | 0.00495 | 11:04 | 46:01:01 | 09:01:02 | 1.00 | 0.00495 |
| 24:20:00 | 08:01:01 | 15:02:01 | 1.00 | 0.00495 | 11:04 | 51:02:01 | 15:02:01 | 1.00 | 0.00495 |
| 26:01:01 | 01:02:01 | 15:11:01 | 1.00 | 0.00495 | 24:02:01 | 13:01:01 | 13:12:01 | 1.00 | 0.00495 |
| 26:01:01 | 07:02:01 | 38:02:01 | 1.00 | 0.00495 | 24:02:01 | 15:02:01 | 15:02:01 | 1.00 | 0.00495 |
| 26:01:01 | 08:01:01 | 40:06:01 | 1.00 | 0.00495 | 24:02:01 | 15:25:01 | 04:03:01 | 1.00 | 0.00495 |
| 26:01:01 | 01:02:01 | 46:01:01 | 1.00 | 0.00495 | 24:02:01 | 18:01:01 | 15:02:01 | 1.00 | 0.00495 |
| 29:01:01 | 03:03:01 | 55:02:01 | 1.00 | 0.00495 | 24:02:01 | 35:01:01 | 04:03:01 | 1.00 | 0.00495 |
| 30:01:01 | 06:02:01 | 13:02:01 | 1.00 | 0.00495 | 24:02:01 | 35:03:01 | 13:01:01 | 1.00 | 0.00495 |
| 31:01:02 | 01:02:01 | 54:01:01 | 1.00 | 0.00495 | 24:02:01 | 40:01:02 | 12:02:01 | 1.00 | 0.00495 |
| 32:01:01 | 07:02:01 | 07:02:01 | 1.00 | 0.00495 | 24:02:01 | 40:06:01 | 09:01:02 | 1.00 | 0.00495 |
| 33:01:01 | 01:02:01 | 46:01:01 | 1.00 | 0.00495 | 24:02:01 | 40:06:01 | 12:02:01 | 1.00 | 0.00495 |
| 33:01:01 | 03:02:02 | 58:01:01 | 1.00 | 0.00495 | 24:02:01 | 46:01:01 | 12:02:01 | 1.00 | 0.00495 |
| 33:03:01 | 07:02:01 | 07:02:01 | 1.00 | 0.00495 | 24:02:01 | 51:01:01 | 12:02:01 | 1.00 | 0.00495 |
| 33:03:01 | 07:02:01 | 13:01:01 | 1.00 | 0.00495 | 24:02:13 | 52:01:01 | 15:02:02 | 1.00 | 0.00495 |
| 33:03:01 | 07:06 | 15:12 | 1.00 | 0.00495 | 24:02:40 | 35:05:01 | 12:02:01 | 1.00 | 0.00495 |
| 33:03:01 | 04:01:01 | 15:27:01 | 1.00 | 0.00495 | 24:03:01 | 15:25:01 | 15:02:01 | 1.00 | 0.00495 |
| 33:03:01 | 07:02:01 | 39:01:01 | 1.00 | 0.00495 | 24:03:01 | 44:03:02 | 14:18 | 1.00 | 0.00495 |
| 33:03:01 | 03:04:01 | 40:01:02 | 1.00 | 0.00495 | 24:07:01 | 35:05:01 | 12:02:01 | 1.00 | 0.00495 |
| 33:03:01 | 12:02:02 | 52:01:01 | 1.00 | 0.00495 | 24:07:01 | 40:06:01 | 12:02:01 | 1.00 | 0.00495 |
| 34:01:01 | 04:03:01 | 15:25:01 | 1.00 | 0.00495 | 24:10:01 | 18:02 | 12:02:01 | 1.00 | 0.00495 |
| 34:01:01 | 04:03:01 | 56:01:01 | 1.00 | 0.00495 | 24:20:00 | 07:02:01 | 15:02:01 | 1.00 | 0.00495 |
| 34:01:01 | 07:02:01 | 56:01:01 | 1.00 | 0.00495 | 24:20:00 | 15:02:01 | 12:02:01 | 1.00 | 0.00495 |
| 68:01:02 | 08:01:01 | 40:06:01 | 1.00 | 0.00495 | 24:20:00 | 48:01:01 | 10:01:01 | 1.00 | 0.00495 |
| 74:02:01 | 04:01:01 | 15:01:01 | 1.00 | 0.00495 | 26:01:01 | 15:11:01 | 09:01:02 | 1.00 | 0.00495 |
|  |  |  |  |  | 26:01:01 | 38:02:01 | 13:02:01 | 1.00 | 0.00495 |
|  |  |  |  |  | 26:01:01 | 40:06:01 | 04:01:01 | 1.00 | 0.00495 |
|  |  |  |  |  | 26:01:01 | 46:01:01 | 12:02:01 | 1.00 | 0.00495 |
|  |  |  |  |  | 29:01:01 | 15:02:01 | 13:12:01 | 1.00 | 0.00495 |
|  |  |  |  |  | 29:01:01 | 55:02:01 | 12:02:01 | 1.00 | 0.00495 |
|  |  |  |  |  | 30:01:01 | 13:02:01 | 03:01:01 | 1.00 | 0.00495 |
|  |  |  |  |  | 31:01:02 | 48:01:01 | 09:01:02 | 1.00 | 0.00495 |
|  |  |  |  |  | 32:01:01 | 13:01:01 | 12:02:01 | 1.00 | 0.00495 |
|  |  |  |  |  | 33:01:01 | 13:01:01 | 04:05:01 | 1.00 | 0.00495 |
|  |  |  |  |  | 33:01:01 | 46:01:01 | 09:01:02 | 1.00 | 0.00495 |
|  |  |  |  |  | 33:03:01 | 13:01:01 | 16:02:01 | 1.00 | 0.00495 |
|  |  |  |  |  | 33:03:01 | 15:12 | 07:01:01 | 1.00 | 0.00495 |
|  |  |  |  |  | 33:03:01 | 15:13:01 | 10:01:01 | 1.00 | 0.00495 |
|  |  |  |  |  | 33:03:01 | 15:27:01 | 04:06:01 | 1.00 | 0.00495 |
|  |  |  |  |  | 33:03:01 | 39:01:01 | 04:05:01 | 1.00 | 0.00495 |
|  |  |  |  |  | 33:03:01 | 40:01:02 | 15:01:01 | 1.00 | 0.00495 |
|  |  |  |  |  | 33:03:01 | 48:01:01 | 09:01:02 | 1.00 | 0.00495 |
|  |  |  |  |  | 33:03:01 | 51:06:01 | 10:01:01 | 1.00 | 0.00495 |
|  |  |  |  |  | 33:03:01 | 56:04:00 | 04:05:01 | 1.00 | 0.00495 |
|  |  |  |  |  | 33:03:01 | 56:04:00 | 12:02:01 | 1.00 | 0.00495 |
|  |  |  |  |  | 33:03:01 | 58:01:01 | 11:06:01 | 1.00 | 0.00495 |
|  |  |  |  |  | 33:03:01 | 58:01:01 | 12:02:01 | 1.00 | 0.00495 |
|  |  |  |  |  | 34:01:01 | 15:35 | 15:02:01 | 1.00 | 0.00495 |
|  |  |  |  |  | 68:01:02 | 46:01:01 | 15:02:01 | 1.00 | 0.00495 |
|  |  |  |  |  | 74:02:01 | 38:02:01 | 04:06:01 | 1.00 | 0.00495 |
