# Supplementary material for: High-Resolution HLA Typing of HLA-A, -B, -C, -DRB1, and -DQB1 in Kinh Vietnamese by Using Next-Generation Sequencing
Source: Front Genet. 2020 Apr 30;11:383. doi: 10.3389/fgene.2020.00383 (PMC7204072; doi:10.3389/fgene.2020.00383)
Supplement: Supplementary file 3 [file Table_3.DOCX]

Supplementary table 3. Haplotype frequencies of five-locus HLA

| A | C | B | DRB1 | DQB1 | Est. count | hap.freq |
| --- | --- | --- | --- | --- | --- | --- |
| 29:01:01 | 15:05:02 | 07:05:01 | 10:01:01 | 05:01:01 | 9.00 | 0.04455 |
| 33:03:01 | 03:02:02 | 58:01:01 | 03:01:01 | 02:01:01 | 9.00 | 0.04455 |
| 11:01:01 | 08:01:01 | 15:02:01 | 12:02:01 | 03:01:01 | 7.77 | 0.03844 |
| 02:07:01 | 01:02:01 | 46:01:01 | 09:01:02 | 03:03:02 | 5.77 | 0.02854 |
| 11:01:01 | 01:02:01 | 46:01:01 | 09:01:02 | 03:03:02 | 4.23 | 0.02096 |
| 02:03:01 | 07:02:01 | 38:02:01 | 08:03:02 | 06:01:01 | 4.00 | 0.01980 |
| 02:07:01 | 08:01:01 | 15:02:01 | 12:02:01 | 03:01:01 | 3.23 | 0.01601 |
| 11:01:01 | 07:02:01 | 40:01:02 | 12:02:01 | 03:01:01 | 3.00 | 0.01485 |
| 24:07:01 | 04:01:01 | 35:05:01 | 12:02:01 | 03:01:01 | 3.00 | 0.01485 |
| 29:01:01 | 15:05:02 | 07:05:01 | 09:01:02 | 03:03:02 | 3.00 | 0.01485 |
| 02:01:01 | 03:03:01 | 35:01:01 | 07:01:01 | 02:02:01 | 2.00 | 0.00990 |
| 02:03:01 | 07:02:01 | 38:02:01 | 12:02:01 | 05:02:01 | 2.00 | 0.00990 |
| 02:07:01 | 08:01:01 | 15:02:01 | 13:12:01 | 03:01:01 | 2.00 | 0.00990 |
| 02:07:01 | 01:02:01 | 46:01:01 | 12:02:01 | 03:01:01 | 2.00 | 0.00990 |
| 11:01:01 | 07:02:01 | 07:02:01 | 10:01:01 | 05:01:01 | 2.00 | 0.00990 |
| 11:01:01 | 08:01:01 | 15:02:01 | 07:01:01 | 02:02:01 | 2.00 | 0.00990 |
| 11:01:01 | 07:02:01 | 15:25:01 | 15:02:01 | 03:01:01 | 2.00 | 0.00990 |
| 11:01:01 | 07:02:01 | 38:02:01 | 15:02:01 | 05:01:01 | 2.00 | 0.00990 |
| 11:01:01 | 07:02:01 | 39:01:01 | 14:54:01 | 03:01:01 | 2.00 | 0.00990 |
| 11:01:01 | 03:02:02 | 58:01:01 | 03:01:01 | 02:01:01 | 2.00 | 0.00990 |
| 24:02:01 | 03:04:01 | 13:01:01 | 16:02:01 | 05:02:01 | 2.00 | 0.00990 |
| 24:02:01 | 07:02:01 | 15:02:01 | 12:02:01 | 03:01:01 | 2.00 | 0.00990 |
| 24:02:01 | 03:04:01 | 27:06:00 | 12:02:01 | 03:01:01 | 2.00 | 0.00990 |
| 24:02:01 | 04:01:01 | 35:05:01 | 08:03:02 | 06:01:01 | 2.00 | 0.00990 |
| 29:01:01 | 08:01:01 | 15:02:01 | 12:02:01 | 03:01:01 | 2.00 | 0.00990 |
| 31:01:02 | 15:02:01 | 51:02:01 | 08:03:02 | 06:01:01 | 2.00 | 0.00990 |
| 33:03:01 | 01:02:01 | 56:04:00 | 12:02:01 | 03:01:01 | 2.00 | 0.00990 |
| 01:01:01 | 07:01:02 | 15:17:01 | 13:02:01 | 06:04:01 | 1.00 | 0.00495 |
| 01:01:01 | 06:02:01 | 57:01:01 | 07:01:01 | 03:03:02 | 1.00 | 0.00495 |
| 01:01:01 | 03:02:02 | 58:01:01 | 13:02:01 | 06:09:01 | 1.00 | 0.00495 |
| 02:01:01 | 04:03:01 | 40:01:02 | 09:01:02 | 03:03:02 | 1.00 | 0.00495 |
| 02:01:01 | 12:02:02 | 52:01:01 | 15:02:01 | 03:03:02 | 1.00 | 0.00495 |
| 02:01:01 | 01:02:01 | 54:01:01 | 09:01:02 | 03:03:02 | 1.00 | 0.00495 |
| 02:01:01 | 04:03:01 | 55:02:01 | 15:01:01 | 06:01:01 | 1.00 | 0.00495 |
| 02:03:01 | 03:04:01 | 27:06:00 | 12:02:01 | 05:02:02 | 1.00 | 0.00495 |
| 02:03:01 | 07:02:01 | 38:02:01 | 04:03:01 | 03:02:01 | 1.00 | 0.00495 |
| 02:03:01 | 07:02:01 | 38:02:01 | 11:01:01 | 03:01:01 | 1.00 | 0.00495 |
| 02:03:01 | 07:02:01 | 39:09:01 | 15:02:01 | 05:02:01 | 1.00 | 0.00495 |
| 02:03:01 | 03:17 | 40:01:02 | 09:01:02 | 06:01:01 | 1.00 | 0.00495 |
| 02:03:01 | 04:03:01 | 40:01:02 | 12:02:01 | 03:01:01 | 1.00 | 0.00495 |
| 02:03:01 | 04:03:01 | 46:01:01 | 04:05:01 | 04:01:01 | 1.00 | 0.00495 |
| 02:03:01 | 07:02:01 | 51:01:01 | 09:01:02 | 05:03:01 | 1.00 | 0.00495 |
| 02:03:01 | 04:03:01 | 54:01:01 | 04:05:01 | 04:01:01 | 1.00 | 0.00495 |
| 02:03:02 | 07:02:01 | 54:01:01 | 14:04:01 | 05:01:03 | 1.00 | 0.00495 |
| 02:06:01 | 07:02:01 | 15:01:01 | 11:06:01 | 05:18 | 1.00 | 0.00495 |
| 02:06:01 | 08:01:01 | 15:25:01 | 16:02:01 | 05:02:01 | 1.00 | 0.00495 |
| 02:06:01 | 03:03:01 | 35:01:01 | 08:03:02 | 06:02:01 | 1.00 | 0.00495 |
| 02:06:01 | 04:03:01 | 38:02:01 | 15:02:01 | 05:02:01 | 1.00 | 0.00495 |
| 02:06:01 | 03:04:01 | 40:01:02 | 12:02:01 | 03:01:01 | 1.00 | 0.00495 |
| 02:06:01 | 12:02:02 | 52:01:01 | 09:01:02 | 05:01:01 | 1.00 | 0.00495 |
| 02:07:01 | 07:02:01 | 07:02:01 | 14:54:01 | 05:03:01 | 1.00 | 0.00495 |
| 02:07:01 | 07:02:01 | 13:01:01 | 16:02:01 | 05:02:01 | 1.00 | 0.00495 |
| 02:07:01 | 01:02:01 | 15:12 | 12:02:01 | 03:01:01 | 1.00 | 0.00495 |
| 02:07:01 | 07:02:01 | 18:01:01 | 03:01:01 | 02:01:01 | 1.00 | 0.00495 |
| 02:07:01 | 03:04:02 | 27:06:00 | 03:01:01 | 03:01:01 | 1.00 | 0.00495 |
| 02:07:01 | 01:02:01 | 46:01:01 | 10:01:01 | 05:01:01 | 1.00 | 0.00495 |
| 02:07:01 | 01:02:01 | 51:01:01 | 11:01:01 | 03:03:02 | 1.00 | 0.00495 |
| 03:01:01 | 03:04:01 | 40:01:02 | 14:05:01 | 05:03:01 | 1.00 | 0.00495 |
| 03:02:01 | 06:02:01 | 13:02:01 | 04:05:01 | 04:01:01 | 1.00 | 0.00495 |
| 03:02:01 | 08:01:01 | 38:02:01 | 15:02:01 | 05:01:01 | 1.00 | 0.00495 |
| 11:01:01 | 07:04:01 | 8:01:01 | 11:06:01 | 03:01:01 | 1.00 | 0.00495 |
| 11:01:01 | 03:04:01 | 13:01:01 | 14:10 | 05:10 | 1.00 | 0.00495 |
| 11:01:01 | 03:03:01 | 15:12 | 12:02:01 | 03:01:01 | 1.00 | 0.00495 |
| 11:01:01 | 04:03:01 | 15:25:01 | 10:01:01 | 05:18 | 1.00 | 0.00495 |
| 11:01:01 | 04:03:01 | 15:25:01 | 13:12:01 | 03:01:01 | 1.00 | 0.00495 |
| 11:01:01 | 04:03:01 | 15:25:01 | 16:02:01 | 05:02:01 | 1.00 | 0.00495 |
| 11:01:01 | 04:82 | 15:25:01 | 12:02:01 | 03:01:01 | 1.00 | 0.00495 |
| 11:01:01 | 07:01:01 | 37:01:01 | 15:02:01 | 05:01:01 | 1.00 | 0.00495 |
| 11:01:01 | 07:02:01 | 38:02:01 | 04:06:01 | 03:03:02 | 1.00 | 0.00495 |
| 11:01:01 | 07:02:01 | 38:02:01 | 08:12 | 06:01:01 | 1.00 | 0.00495 |
| 11:01:01 | 03:03:01 | 40:01:02 | 15:01:01 | 06:01:01 | 1.00 | 0.00495 |
| 11:01:01 | 03:04:01 | 40:01:02 | 04:05:01 | 04:01:01 | 1.00 | 0.00495 |
| 11:01:01 | 07:02:01 | 40:01:02 | 11:01:01 | 05:02:01 | 1.00 | 0.00495 |
| 11:01:01 | 07:02:01 | 40:02:01 | 04:05:01 | 04:01:01 | 1.00 | 0.00495 |
| 11:01:01 | 03:04:01 | 44:03:02 | 13:12:01 | 03:01:01 | 1.00 | 0.00495 |
| 11:01:01 | 14:02:01 | 51:06:01 | 16:02:01 | 05:02:01 | 1.00 | 0.00495 |
| 11:01:01 | 14:02:01 | 55:02:01 | 14:05:01 | 05:03:02 | 1.00 | 0.00495 |
| 11:01:01 | 07:02:01 | 55:18:00 | 04:05:01 | 04:01:01 | 1.00 | 0.00495 |
| 11:01:01 | 01:02:01 | 56:01:01 | 04:05:01 | 04:01:01 | 1.00 | 0.00495 |
| 11:02:01 | 07:02:01 | 15:25:01 | 12:02:01 | 03:01:01 | 1.00 | 0.00495 |
| 11:02:01 | 07:02:01 | 15:35 | 15:02:01 | 05:02:01 | 1.00 | 0.00495 |
| 11:02:01 | 04:03:01 | 39:01:01 | 15:01:01 | 06:02:01 | 1.00 | 0.00495 |
| 11:02:01 | 07:02:01 | 40:01:02 | 08:03:02 | 06:01:01 | 1.00 | 0.00495 |
| 11:02:01 | 07:02:01 | 40:01:02 | 15:01:01 | 06:01:01 | 1.00 | 0.00495 |
| 11:04 | 01:02:01 | 46:01:01 | 09:01:02 | 03:03:02 | 1.00 | 0.00495 |
| 11:04 | 15:02:01 | 51:02:01 | 15:02:01 | 05:01:01 | 1.00 | 0.00495 |
| 24:02:01 | 03:04:01 | 15:02:01 | 04:05:01 | 04:01:01 | 1.00 | 0.00495 |
| 24:02:01 | 08:01:01 | 15:02:01 | 15:02:01 | 05:02:01 | 1.00 | 0.00495 |
| 24:02:01 | 04:03:01 | 15:25:01 | 15:02:01 | 05:01:01 | 1.00 | 0.00495 |
| 24:02:01 | 07:02:01 | 15:25:01 | 04:05:01 | 05:02:01 | 1.00 | 0.00495 |
| 24:02:01 | 01:02:01 | 18:01:01 | 15:02:01 | 05:03:11 | 1.00 | 0.00495 |
| 24:02:01 | 03:03:01 | 35:01:01 | 04:03:01 | 03:02:01 | 1.00 | 0.00495 |
| 24:02:01 | 04:01:01 | 35:03:01 | 13:01:01 | 06:03:01 | 1.00 | 0.00495 |
| 24:02:01 | 04:03:01 | 38:02:01 | 11:129 | 05:02:01 | 1.00 | 0.00495 |
| 24:02:01 | 03:03:01 | 40:01:02 | 16:02:01 | 03:01:01 | 1.00 | 0.00495 |
| 24:02:01 | 04:03:01 | 40:01:02 | 04:03:01 | 03:02:01 | 1.00 | 0.00495 |
| 24:02:01 | 03:04:01 | 40:06:01 | 08:03:02 | 06:01:01 | 1.00 | 0.00495 |
| 24:02:01 | 08:01:01 | 40:06:01 | 12:02:01 | 03:01:01 | 1.00 | 0.00495 |
| 24:02:01 | 01:02:01 | 46:01:01 | 09:01:02 | 03:03:02 | 1.00 | 0.00495 |
| 24:02:01 | 01:02:01 | 46:01:01 | 12:02:01 | 05:02:01 | 1.00 | 0.00495 |
| 24:02:01 | 14:02:01 | 51:01:01 | 12:02:01 | 03:01:01 | 1.00 | 0.00495 |
| 24:02:01 | 07:02:01 | 52:01:01 | 13:12:01 | 03:01:01 | 1.00 | 0.00495 |
| 24:02:01 | 01:02:01 | 55:02:01 | 15:02:01 | 05:02:02 | 1.00 | 0.00495 |
| 24:02:01 | 03:02:02 | 58:01:01 | 12:02:01 | 02:01:01 | 1.00 | 0.00495 |
| 24:02:13 | 12:02:02 | 52:01:01 | 15:02:02 | 06:01:01 | 1.00 | 0.00495 |
| 24:02:40 | 04:01:01 | 35:05:01 | 12:02:01 | 03:01:01 | 1.00 | 0.00495 |
| 24:03:01 | 07:06 | 15:12 | 07:01:01 | 05:03:01 | 1.00 | 0.00495 |
| 24:03:01 | 04:03:01 | 15:25:01 | 15:02:01 | 05:01:03 | 1.00 | 0.00495 |
| 24:07:01 | 06:02:01 | 15:02:01 | 09:01:02 | 03:05:02 | 1.00 | 0.00495 |
| 24:07:01 | 08:01:01 | 15:02:01 | 09:01:02 | 05:01:12 | 1.00 | 0.00495 |
| 24:07:01 | 04:01:01 | 35:05:01 | 15:02:01 | 03:03:05 | 1.00 | 0.00495 |
| 24:10:01 | 07:04:01 | 18:02 | 16:02:01 | 05:02:01 | 1.00 | 0.00495 |
| 24:20:00 | 08:01:01 | 15:02:01 | 12:02:01 | 03:01:01 | 1.00 | 0.00495 |
| 24:20:00 | 08:03:01 | 48:01:01 | 10:01:01 | 05:01:01 | 1.00 | 0.00495 |
| 24:20:00 | 08:03:01 | 48:01:01 | 15:02:01 | 03:03:02 | 1.00 | 0.00495 |
| 26:01:01 | 01:02:01 | 15:11:01 | 12:02:01 | 03:01:01 | 1.00 | 0.00495 |
| 26:01:01 | 07:02:01 | 38:02:01 | 13:02:01 | 06:09:01 | 1.00 | 0.00495 |
| 26:01:01 | 08:01:01 | 40:06:01 | 04:01:01 | 03:01:01 | 1.00 | 0.00495 |
| 26:01:01 | 01:02:01 | 46:01:01 | 09:01:02 | 03:03:02 | 1.00 | 0.00495 |
| 29:01:01 | 15:05:02 | 07:05:01 | 11:01:01 | 03:01:01 | 1.00 | 0.00495 |
| 29:01:01 | 15:05:02 | 07:05:01 | 13:12:01 | 03:01:01 | 1.00 | 0.00495 |
| 29:01:01 | 03:03:01 | 55:02:01 | 09:01:02 | 03:03:02 | 1.00 | 0.00495 |
| 30:01:01 | 06:02:01 | 13:02:01 | 03:01:01 | 02:01:01 | 1.00 | 0.00495 |
| 31:01:02 | 08:01:01 | 48:01:01 | 11:01:01 | 03:01:01 | 1.00 | 0.00495 |
| 32:01:01 | 03:04:01 | 13:01:01 | 12:02:01 | 03:01:01 | 1.00 | 0.00495 |
| 33:01:01 | 03:04:01 | 13:01:01 | 04:05:01 | 04:01:01 | 1.00 | 0.00495 |
| 33:01:01 | 01:02:01 | 46:01:01 | 09:01:02 | 03:03:02 | 1.00 | 0.00495 |
| 33:03:01 | 07:02:01 | 07:02:01 | 09:01:02 | 06:01:01 | 1.00 | 0.00495 |
| 33:03:01 | 03:02:02 | 15:13:01 | 10:01:01 | 03:03:02 | 1.00 | 0.00495 |
| 33:03:01 | 03:02:02 | 15:27:01 | 11:06:01 | 03:01:01 | 1.00 | 0.00495 |
| 33:03:01 | 07:02:01 | 39:01:01 | 04:05:01 | 04:01:01 | 1.00 | 0.00495 |
| 33:03:01 | 03:04:01 | 40:01:02 | 15:01:01 | 05:02:01 | 1.00 | 0.00495 |
| 33:03:01 | 03:03:01 | 44:03:02 | 14:18 | 05:03:01 | 1.00 | 0.00495 |
| 33:03:01 | 14:02:01 | 51:01:01 | 16:02:01 | 05:02:01 | 1.00 | 0.00495 |
| 33:03:01 | 03:02:02 | 58:01:01 | 03:01:01 | 05:01:01 | 1.00 | 0.00495 |
| 33:03:01 | 03:02:02 | 58:01:01 | 10:01:01 | 05:01:01 | 1.00 | 0.00495 |
| 33:03:01 | 03:02:02 | 58:01:01 | 12:02:01 | 03:01:01 | 1.00 | 0.00495 |
| 33:03:01 | 04:01:01 | 58:01:01 | 04:06:01 | 03:02:01 | 1.00 | 0.00495 |
| 34:01:01 | 07:02:01 | 15:35 | 15:02:01 | 05:02:04 | 1.00 | 0.00495 |
| 34:01:01 | 04:03:01 | 56:01:01 | 04:05:01 | 04:02:01 | 1.00 | 0.00495 |
| 34:01:01 | 07:02:01 | 56:01:01 | 04:05:01 | 04:02:01 | 1.00 | 0.00495 |
| 68:01:02 | 08:01:01 | 40:06:01 | 15:02:01 | 05:02:01 | 1.00 | 0.00495 |
| 74:02:01 | 04:01:01 | 15:01:01 | 09:01:02 | 03:02:01 | 1.00 | 0.00495 |
