# Supplementary material for: High-Resolution HLA Typing of HLA-A, -B, -C, -DRB1, and -DQB1 in Kinh Vietnamese by Using Next-Generation Sequencing
Source: Front Genet. 2020 Apr 30;11:383. doi: 10.3389/fgene.2020.00383 (PMC7204072; doi:10.3389/fgene.2020.00383)
Supplement: Supplementary file 4 [file Table_4.DOCX]

Supplementary table 4. Haplotype frequencies of ten-locus HLA

| Haplotype | Count | hap. Frequency |
| --- | --- | --- |
| A*02:07:01~A*33:03:01~B*13:01:01~B*51:01:01~C*07:02:01~C*14:02:01~DRB1*16:02:01~DRB1*16:02:01~DQB1*05:02:01~DQB1*05:02:01 | 1 | 0.990 |
| A*11:01:01~A*11:02:01~B*15:02:01~B*15:35~C*07:02:01~C*08:01:01~DRB1*15:02:01~DRB1*12:02:01~DQB1*03:01:01~DQB1*05:02:01 | 1 | 0.990 |
| A*02:01:01~A*02:03:01~B*40:01:02~B*52:01:01~C*03:17~C*12:02:02~DRB1*15:02:01~DRB1*09:01:02~DQB1*03:03:02~DQB1*06:01:01 | 1 | 0.990 |
| A*11:01:01~A*74:02:01~B*15:01:01~B*38:02:01~C*04:01:01~C*07:02:01~DRB1*04:06:01~DRB1*09:01:02~DQB1*03:02:01~DQB1*03:03:02 | 1 | 0.990 |
| A*11:01:01~A*33:01:01~B*13:01:01~B*58:01:01~C*03:02:02~C*03:04:01~DRB1*03:01:01~DRB1*04:05:01~DQB1*02:01:01~DQB1*04:01:01 | 1 | 0.990 |
| A*02:03:02~A*24:02:01~B*18:01:01~B*54:01:01~C*01:02:01~C*07:02:01~DRB1*15:02:01~DRB1*14:04:01~DQB1*05:01:03~DQB1*05:03:11 | 1 | 0.990 |
| A*02:07:01~A*33:03:01~B*46:01:01~B*58:01:01~C*01:02:01~C*03:02:02~DRB1*03:01:01~DRB1*09:01:02~DQB1*02:01:01~DQB1*03:03:02 | 1 | 0.990 |
| A*02:01:01~A*11:01:01~B*40:01:02~B*40:01:02~C*04:03:01~C*07:02:01~DRB1*12:02:01~DRB1*09:01:02~DQB1*03:01:01~DQB1*03:03:02 | 1 | 0.990 |
| A*24:02:01~A*34:01:01~B*13:01:01~B*56:01:01~C*03:04:01~C*07:02:01~DRB1*16:02:01~DRB1*04:05:01~DQB1*04:02:01~DQB1*05:02:01 | 1 | 0.990 |
| A*02:06:01~A*24:02:01~B*15:02:01~B*15:25:01~C*07:02:01~C*08:01:01~DRB1*16:02:01~DRB1*12:02:01~DQB1*03:01:01~DQB1*05:02:01 | 1 | 0.990 |
| A*11:01:01~A*11:01:01~B*15:02:01~B*15:02:01~C*08:01:01~C*08:01:01~DRB1*12:02:01~DRB1*12:02:01~DQB1*03:01:01~DQB1*03:01:01 | 1 | 0.990 |
| A*11:01:01~A*11:01:01~B*40:01:02~B*46:01:01~C*01:02:01~C*03:04:01~DRB1*04:05:01~DRB1*09:01:02~DQB1*03:03:02~DQB1*04:01:01 | 1 | 0.990 |
| A*33:03:01~A*33:03:01~B*15:27:01~B*58:01:01~C*03:02:02~C*04:01:01~DRB1*11:06:01~DRB1*04:06:01~DQB1*03:01:01~DQB1*03:02:01 | 1 | 0.990 |
| A*11:01:01~A*24:02:01~B*15:25:01~B*38:02:01~C*04:03:01~C*07:02:01~DRB1*15:02:01~DRB1*11:129~DQB1*03:01:01~DQB1*05:02:01 | 1 | 0.990 |
| A*11:01:01~A*24:02:01~B*13:01:01~B*46:01:01~C*01:02:01~C*03:04:01~DRB1*16:02:01~DRB1*09:01:02~DQB1*03:03:02~DQB1*05:02:01 | 1 | 0.990 |
| A*26:01:01~A*29:01:01~B*15:11:01~B*55:02:01~C*01:02:01~C*03:03:01~DRB1*12:02:01~DRB1*09:01:02~DQB1*03:01:01~DQB1*03:03:02 | 1 | 0.990 |
| A*11:02:01~A*29:01:01~B*07:05:01~B*15:25:01~C*07:02:01~C*15:05:02~DRB1*10:01:01~DRB1*12:02:01~DQB1*03:01:01~DQB1*05:01:01 | 1 | 0.990 |
| A*02:06:01~A*11:01:01~B*35:01:01~B*40:01:02~C*03:03:01~C*03:03:01~DRB1*15:01:01~DRB1*08:03:02~DQB1*06:01:01~DQB1*06:02:01 | 1 | 0.990 |
| A*11:01:01~A*24:02:01~B*15:02:01~B*58:01:01~C*03:02:02~C*07:02:01~DRB1*03:01:01~DRB1*12:02:01~DQB1*02:01:01~DQB1*03:01:01 | 1 | 0.990 |
| A*26:01:01~A*29:01:01~B*07:05:01~B*40:06:01~C*08:01:01~C*15:05:02~DRB1*10:01:01~DRB1*04:01:01~DQB1*03:01:01~DQB1*05:01:01 | 1 | 0.990 |
| A*11:01:01~A*30:01:01~B*13:02:01~B*15:02:01~C*06:02:01~C*08:01:01~DRB1*03:01:01~DRB1*07:01:01~DQB1*02:01:01~DQB1*02:02:01 | 1 | 0.990 |
| A*02:07:01~A*24:02:01~B*46:01:01~B*51:01:01~C*01:02:01~C*14:02:01~DRB1*12:02:01~DRB1*12:02:01~DQB1*03:01:01~DQB1*03:01:01 | 1 | 0.990 |
| A*11:01:01~A*33:03:01~B*15:25:01~B*58:01:01~C*03:02:02~C*04:03:01~DRB1*03:01:01~DRB1*13:12:01~DQB1*02:01:01~DQB1*03:01:01 | 1 | 0.990 |
| A*24:07:01~A*26:01:01~B*35:05:01~B*46:01:01~C*01:02:01~C*04:01:01~DRB1*12:02:01~DRB1*09:01:02~DQB1*03:01:01~DQB1*03:03:02 | 1 | 0.990 |
| A*29:01:01~A*33:01:01~B*15:02:01~B*46:01:01~C*01:02:01~C*08:01:01~DRB1*12:02:01~DRB1*09:01:02~DQB1*03:01:01~DQB1*03:03:02 | 1 | 0.990 |
| A*02:07:01~A*11:01:01~B*15:02:01~B*46:01:01~C*01:02:01~C*08:01:01~DRB1*13:12:01~DRB1*09:01:02~DQB1*03:01:01~DQB1*03:03:02 | 1 | 0.990 |
| A*11:01:01~A*24:20~B*48:01:01~B*55:18~C*07:02:01~C*08:03:01~DRB1*10:01:01~DRB1*04:05:01~DQB1*04:01:01~DQB1*05:01:01 | 1 | 0.990 |
| A*01:01:01~A*11:01:01~B*15:17:01~B*46:01:01~C*01:02:01~C*07:01:02~DRB1*13:02:01~DRB1*09:01:02~DQB1*03:03:02~DQB1*06:04:01 | 1 | 0.990 |
| A*11:01:01~A*32:01:01~B*07:02:01~B*13:01:01~C*03:04:01~C*07:02:01~DRB1*10:01:01~DRB1*12:02:01~DQB1*03:01:01~DQB1*05:01:01 | 1 | 0.990 |
| A*29:01:01~A*33:03:01~B*07:05:01~B*56:04~C*01:02:01~C*15:05:02~DRB1*10:01:01~DRB1*12:02:01~DQB1*03:01:01~DQB1*05:01:01 | 1 | 0.990 |
| A*02:03:01~A*24:02:13~B*38:02:01~B*52:01:01~C*07:02:01~C*12:02:02~DRB1*15:02:02~DRB1*04:03:01~DQB1*03:02:01~DQB1*06:01:01 | 1 | 0.990 |
| A*11:01:01 ~A*11:01:01~B*15:02:01~B*15:25:01~C*04:03:01~C*08:01:01~DRB1*16:02:01~DRB1*12:02:01~DQB1*03:01:01~DQB1*05:02:01 | 1 | 0.990 |
| A*01:01:01~A*29:01:01~B*07:05:01~B*57:01:01~C*06:02:01~C*15:05:02~DRB1*07:01:01~DRB1*09:01:02~DQB1*03:03:02~DQB1*03:03:02 | 1 | 0.990 |
| A*02:03:01~A*29:01:01~B*07:05:01~B*38:02:01~C*07:02:01~C*15:05:02~DRB1*08:03:02~DRB1*10:01:01~DQB1*05:01:01~DQB1*06:01:01 | 1 | 0.990 |
| A*02:06:01~A*24:02:01~B*35:05:01~B*40:01:02~C*03:04:01~C*04:01:01~DRB1*08:03:02~DRB1*12:02:01~DQB1*03:01:01~DQB1*06:01:01 | 1 | 0.990 |
| A*24:03:01~A*34:01:01~B*15:25:01~B*15:35~C*04:03:01~C*07:02:01~DRB1*15:02:01~DRB1*15:02:01~DQB1*05:01:03~DQB1*05:02:04 | 1 | 0.990 |
| A*02:03:01~A*24:02:01~B*15:25:01~B*38:02:01~C*04:03:01~C*07:02:01~DRB1*15:02:01~DRB1*08:03:02~DQB1*05:01:01~DQB1*06:01:01 | 1 | 0.990 |
| A*02:07:01~A*29:01:01~B*07:05:01~B*46:01:01~C*01:02:01~C*15:05:02~DRB1*11:01:01~DRB1*09:01:02~DQB1*03:01:01~DQB1*03:03:02 | 1 | 0.990 |
| A*02:07:01~A*24:02:01~B*27:06~B*58:01:01~C*03:02:02~C*03:04:02~DRB1*03:01:01~DRB1*12:02:01~DQB1*02:01:01~DQB1*03:01:01 | 1 | 0.990 |
| A*24:02:01~A*68:01:02~B*40:06:01~B*46:01:01~C*01:02:01~C*08:01:01~DRB1*15:02:01~DRB1*09:01:02~DQB1*03:03:02~DQB1*05:02:01 | 1 | 0.990 |
| A*02:06:01~A*24:02:01~B*15:25:01~B*38:02:01~C*04:03:01~C*07:02:01~DRB1*15:02:01~DRB1*04:05:01~DQB1*05:02:01~DQB1*05:02:01 | 1 | 0.990 |
| A*02:03:01~A*24:10:01~B*18:02~B*38:02:01~C*07:02:01~C*07:04:01~DRB1*16:02:01~DRB1*12:02:01~DQB1*05:02:01~DQB1*05:02:01 | 1 | 0.990 |
| A*24:02:01~A*24:07:01~B*35:05:01~B*40:06:01~C*03:04:01~C*04:01:01~DRB1*08:03:02~DRB1*12:02:01~DQB1*03:01:01~DQB1*06:01:01 | 1 | 0.990 |
| A*02:03:01~A*11:01:01~B*51:01:01~B*55:02:01~C*07:02:01~C*14:02:01~DRB1*14:05:01~DRB1*09:01:02~DQB1*05:03:01~DQB1*05:03:02 | 1 | 0.990 |
| A*24:02:40~A*33:03:01~B*35:05:01~B*58:01:01~C*03:02:02~C*04:01:01~DRB1*03:01:01~DRB1*12:02:01~DQB1*02:01:01~DQB1*03:01:01 | 1 | 0.990 |
| A*11:01:01~A*33:03:01~B*51:06:01~B*58:01:01~C*03:02:02~C*14:02:01~DRB1*16:02:01~DRB1*10:01:01~DQB1*05:01:01~DQB1*05:02:01 | 1 | 0.990 |
| A*02:03:01~A*33:03:01~B*38:02:01~B*40:01:02~C*03:04:01~C*07:02:01~DRB1*15:01:01~DRB1*08:03:02~DQB1*05:02:01~DQB1*06:01:01 | 1 | 0.990 |
| A*11:01:01~A*11:01:01~B*15:02:01~B*44:03:02~C*03:04:01~C*08:01:01~DRB1*13:12:01~DRB1*07:01:01~DQB1*02:02:01~DQB1*03:01:01 | 1 | 0.990 |
| A*11:01:01~A*24:02:01~B*39:01:01~B*46:01:01~C*01:02:01~C*07:02:01~DRB1*12:02:01~DRB1*14:54:01~DQB1*03:01:01~DQB1*05:02:01 | 1 | 0.990 |
| A*02:07:01~A*11:01:01~B*37:01:01~B*46:01:01~C*01:02:01~C*07:01:01~DRB1*15:02:01~DRB1*10:01:01~DQB1*05:01:01~DQB1*05:01:01 | 1 | 0.990 |
| A*02:07:01~A*11:01:01~B*08:01:01~B*18:01:01~C*07:02:01~C*07:04:01~DRB1*03:01:01~DRB1*11:06:01~DQB1*02:01:01~DQB1*03:01:01 | 1 | 0.990 |
| A*02:01:01~A*29:01:01~B*07:05:01~B*55:02:01~C*04:03:01~C*15:05:02~DRB1*15:01:01~DRB1*10:01:01~DQB1*05:01:01~DQB1*06:01:01 | 1 | 0.990 |
| A*24:03:01~A*33:03:01~B*15:12~B*44:03:02~C*03:03:01~C*07:06~DRB1*14:18~DRB1*07:01:01~DQB1*05:03:01~DQB1*05:03:01 | 1 | 0.990 |
| A*26:01:01~A*33:03:01~B*38:02:01~B*58:01:01~C*03:02:02~C*07:02:01~DRB1*03:01:01~DRB1*13:02:01~DQB1*02:01:01~DQB1*06:09:01 | 1 | 0.990 |
| A*24:02:01~A*33:03:01~B*40:06:01~B*58:01:01~C*03:02:02~C*08:01:01~DRB1*03:01:01~DRB1*12:02:01~DQB1*02:01:01~DQB1*03:01:01 | 1 | 0.990 |
| A*02:06:01~A*11:01:01~B*15:01:01~B*15:25:01~C*04:03:01~C*07:02:01~DRB1*10:01:01~DRB1*11:06:01~DQB1*05:18~DQB1*05:18 | 1 | 0.990 |
| A*02:03:01~A*24:02:01~B*27:06~B*55:02:01~C*01:02:01~C*03:04:01~DRB1*15:02:01~DRB1*12:02:01~DQB1*05:02:02~DQB1*05:02:02 | 1 | 0.990 |
| A*11:01:01~A*24:02:01~B*15:25:01~B*40:01:02~C*04:03:01~C*04:82~DRB1*12:02:01~DRB1*04:03:01~DQB1*03:01:01~DQB1*03:02:01 | 1 | 0.990 |
| A*24:02:01~A*33:03:01~B*15:02:01~B*56:04~C*01:02:01~C*03:04:01~DRB1*12:02:01~DRB1*04:05:01~DQB1*03:01:01~DQB1*04:01:01 | 1 | 0.990 |
| A*11:01:01~A*34:01:01~B*40:01:02~B*56:01:01~C*04:03:01~C*07:02:01~DRB1*12:02:01~DRB1*04:05:01~DQB1*03:01:01~DQB1*04:02:01 | 1 | 0.990 |
| A*02:01:01~A*03:02:01~B*13:02:01~B*35:01:01~C*03:03:01~C*06:02:01~DRB1*04:05:01~DRB1*07:01:01~DQB1*02:02:01~DQB1*04:01:01 | 1 | 0.990 |
| A*24:20~A*33:03:01~B*15:02:01~B*58:01:01~C*03:02:02~C*08:01:01~DRB1*03:01:01~DRB1*12:02:01~DQB1*02:01:01~DQB1*03:01:01 | 1 | 0.990 |
| A*02:03:01~A*24:02:01~B*15:02:01~B*38:02:01~C*07:02:01~C*08:01:01~DRB1*15:02:01~DRB1*08:03:02~DQB1*05:02:01~DQB1*06:01:01 | 1 | 0.990 |
| A*02:03:01~A*31:01:02~B*40:01:02~B*51:02:01~C*04:03:01~C*15:02:01~DRB1*08:03:02~DRB1*12:02:01~DQB1*03:01:01~DQB1*06:01:01 | 1 | 0.990 |
| A*02:03:01~A*11:01:01~B*54:01:01~B*56:01:01~C*01:02:01~C*04:03:01~DRB1*04:05:01~DRB1*04:05:01~DQB1*04:01:01~DQB1*04:01:01 | 1 | 0.990 |
| A*11:01:01~A*24:02:01~B*15:02:01~B*35:03:01~C*04:01:01~C*08:01:01~DRB1*12:02:01~DRB1*13:01:01~DQB1*03:01:01~DQB1*06:03:01 | 1 | 0.990 |
| A*29:01:01~A*33:03:01~B*07:05:01~B*58:01:01~C*03:02:02~C*15:05:02~DRB1*03:01:01~DRB1*10:01:01~DQB1*05:01:01~DQB1*05:01:01 | 1 | 0.990 |
| A*02:03:01~A*02:07:01~B*46:01:01~B*51:01:01~C*01:02:01~C*04:03:01~DRB1*04:05:01~DRB1*11:01:01~DQB1*03:03:02~DQB1*04:01:01 | 1 | 0.990 |
| A*24:07:01 ~A*24:07:01~B*15:02:01~B*35:05:01~C*04:01:01~C*08:01:01~DRB1*15:02:01~DRB1*09:01:02~DQB1*03:03:05~DQB1*05:01:12 | 1 | 0.990 |
| A*02:07:01 ~A*02:07:01~B*46:01:01~B*46:01:01~C*01:02:01~C*01:02:01~DRB1*09:01:02 ~DRB1*09:01:02~DQB1*03:03:02~DQB1*03:03:02 | 1 | 0.990 |
| A*02:07:01~A*29:01:01~B*15:02:01 ~B*15:02:01~C*08:01:01~C*08:01:01~DRB1*12:02:01 ~DRB1*12:02:01~DQB1*03:01:01 ~DQB1*03:01:01 | 1 | 0.990 |
| A*11:01:01~A*29:01:01~B*07:05:01~B*15:12~C*03:03:01~C*15:05:02~DRB1*10:01:01~DRB1*12:02:01~DQB1*03:01:01~DQB1*05:01:01 | 1 | 0.990 |
| A*02:07:01~A*29:01:01~B*07:05:01~B*15:02:01~C*08:01:01~C*15:05:02~DRB1*13:12:01~DRB1*13:12:01~DQB1*03:01:01~DQB1*03:01:01 | 1 | 0.990 |
| A*02:07:01~A*24:02:01~B*15:02:01~B*27:06~C*03:04:01~C*08:01:01~DRB1*12:02:01~DRB1*12:02:01~DQB1*03:01:01~DQB1*03:01:01 | 1 | 0.990 |
| A*01:01:01~A*24:07:01~B*35:05:01~B*58:01:01~C*03:02:02~C*04:01:01~DRB1*12:02:01~DRB1*13:02:01~DQB1*03:01:01~DQB1*06:09:01 | 1 | 0.990 |
| A*02:07:01~A*11:01:01~B*07:02:01~B*38:02:01~C*07:02:01~C*07:02:01~DRB1*15:02:01~DRB1*14:54:01~DQB1*05:01:01~DQB1*05:03:01 | 1 | 0.990 |
| A*11:01:01~A*24:02:01~B*13:01:01~B*52:01:01~C*03:04:01~C*07:02:01~DRB1*13:12:01~DRB1*14:10~DQB1*03:01:01~DQB1*05:10 | 1 | 0.990 |
| A*11:01:01~A*11:01:01~B*39:01:01~B*40:01:02~C*07:02:01~C*07:02:01~DRB1*11:01:01~DRB1*14:54:01~DQB1*03:01:01~DQB1*05:02:01 | 1 | 0.990 |
| A*02:01:01~A*11:01:01~B*15:02:01~B*35:01:01~C*03:03:01~C*08:01:01~DRB1*07:01:01~DRB1*12:02:01~DQB1*02:02:01~DQB1*03:01:01 | 1 | 0.990 |
| A*02:07:01~A*11:01:01~B*15:02:01~B*46:01:01~C*01:02:01~C*08:01:01~DRB1*09:01:02~DRB1*12:02:01~DQB1*03:01:01~DQB1*03:03:02 | 1 | 0.990 |
| A*11:01:01~A*24:02:01~B*35:05:01~B*40:01:02~C*04:01:01~C*07:02:01~DRB1*08:03:02~DRB1*12:02:01~DQB1*03:01:01~DQB1*06:01:01 | 1 | 0.990 |
| A*11:04~A*29:01:01~B*07:05:01~B*51:02:01~C*15:02:01~C*15:05:02~DRB1*10:01:01~DRB1*15:02:01~DQB1*05:01:01~DQB1*05:01:01 | 1 | 0.990 |
| A*11:01:01~A*11:02:01~B*15:25:01~B*39:01:01~C*04:03:01~C*07:02:01~DRB1*15:02:01~DRB1*15:01:01~DQB1*03:01:01~DQB1*06:02:01 | 1 | 0.990 |
| A*02:06:01~A*33:03:01~B*15:13:01~B*52:01:01~C*03:02:02~C*12:02:02~DRB1*09:01:02~DRB1*10:01:01~DQB1*03:03:02~DQB1*05:01:01 | 1 | 0.990 |
| A*02:03:01~A*02:07:01~B*15:02:01~B*39:09:01~C*07:02:01~C*08:01:01~DRB1*12:02:01~DRB1*15:02:01~DQB1*03:01:01~DQB1*05:02:01 | 1 | 0.990 |
| A*24:02:01~A*29:01:01~B*07:05:01~B*35:01:01~C*03:03:01~C*15:05:02~DRB1*04:03:01~DRB1*09:01:02~DQB1*03:02:01~DQB1*03:03:02 | 1 | 0.990 |
| A*11:01:01~A*33:03:01~B*38:02:01~B*58:01:01~C*03:02:02~C*07:02:01~DRB1*12:02:01~DRB1*15:02:01~DQB1*03:01:01~DQB1*05:01:01 | 1 | 0.990 |
| A*24:20~A*33:03:01~B*07:02:01~B*48:01:01~C*07:02:01~C*08:03:01~DRB1*09:01:02~DRB1*15:02:01~DQB1*03:03:02~DQB1*06:01:01 | 1 | 0.990 |
| A*11:01:01~A*11:02:01~B*15:02:01~B*40:01:02~C*07:02:01~C*08:01:01~DRB1*12:02:01~DRB1*15:01:01~DQB1*03:01:01~DQB1*06:01:01 | 1 | 0.990 |
| A*02:03:01~A*11:01:01~B*38:02:01~B*38:02:01~C*07:02:01~C*07:02:01~DRB1*08:12~DRB1*11:01:01~DQB1*03:01:01~DQB1*06:01:01 | 1 | 0.990 |
| A*11:01:01~A*31:01:02~B*40:02:01~B*51:02:01~C*07:02:01~C*15:02:01~DRB1*04:05:01~DRB1*08:03:02~DQB1*04:01:01~DQB1*06:01:01 | 1 | 0.990 |
| A*11:02:01~A*24:02:01~B*27:06~B*40:01:02~C*03:04:01~C*07:02:01~DRB1*08:03:02~DRB1*12:02:01~DQB1*03:01:01~DQB1*06:01:01 | 1 | 0.990 |
| A*02:01:01~A*31:01:02~B*48:01:01~B*54:01:01~C*01:02:01~C*08:01:01~DRB1*09:01:02~DRB1*11:01:01~DQB1*03:01:01~DQB1*03:03:02 | 1 | 0.990 |
| A*02:07:01~A*33:03:01~B*46:01:01~B*58:01:01~C*01:02:01~C*03:02:02~DRB1*03:01:01~DRB1*12:02:01~DQB1*02:01:01~DQB1*03:01:01 | 1 | 0.990 |
| A*11:04~A*29:01:01~B*07:05:01~B*46:01:01~C*01:02:01~C*15:05:02~DRB1*09:01:02~DRB1*10:01:01~DQB1*03:03:02~DQB1*05:01:01 | 1 | 0.990 |
| A*33:03:01~A*33:03:01~B*39:01:01~B*58:01:01~C*03:02:02~C*07:02:01~DRB1*03:01:01~DRB1*04:05:01~DQB1*02:01:01~DQB1*04:01:01 | 1 | 0.990 |
| A*03:01:01~A*11:01:01~B*07:02:01~B*40:01:02~C*03:04:01~C*07:02:01~DRB1*10:01:01~DRB1*14:05:01~DQB1*05:01:01~DQB1*05:03:01 | 1 | 0.990 |
| A*03:02:01~A*24:07:01~B*15:02:01~B*38:02:01~C*06:02:01~C*08:01:01~DRB1*09:01:02~DRB1*15:02:01~DQB1*03:05:02~DQB1*05:01:01 | 1 | 0.990 |
| A*02:03:01~A*24:02:01~B*38:02:01~B*40:01:02~C*03:03:01~C*07:02:01~DRB1*12:02:01~DRB1*16:02:01~DQB1*03:01:01~DQB1*05:02:01 | 1 | 0.990 |
| A*29:01:01~A*33:03:01~B*07:05:01~B*58:01:01~C*03:02:02~C*15:05:02~DRB1*03:01:01~DRB1*09:01:02~DQB1*02:01:01~DQB1*03:03:02 | 1 | 0.990 |
| A*02:07:01~A*02:07:01~B*15:12~B*46:01:01~C*01:02:01~C*01:02:01~DRB1*09:01:02~DRB1*12:02:01~DQB1*03:01:01~DQB1*03:03:02 | 1 | 0.990 |
